# Harmonised statistics and maps of forest biomass and increment in Europe

**DOI:** 10.1038/s41597-023-02868-8

**Published:** 2024-03-06

**Authors:** Valerio Avitabile, Roberto Pilli, Mirco Migliavacca, Gregory Duveiller, Andrea Camia, Viorel Blujdea, Radim Adolt, Iciar Alberdi, Susana Barreiro, Susann Bender, Dragan Borota, Michal Bosela, Olivier Bouriaud, Johannes Breidenbach, Isabel Cañellas, Jura Čavlović, Antoine Colin, Lucio Di Cosmo, Janis Donis, Christoph Fischer, Alexandra Freudenschuss, Jonas Fridman, Patrizia Gasparini, Thomas Gschwantner, Laura Hernández, Kari Korhonen, Gintaras Kulbokas, Vivian Kvist, Nicolas Latte, Andis Lazdins, Philippe Lejeune, Kristaps Makovskis, Gheorghe Marin, Jan Maslo, Artur Michorczyk, Marcin Mionskowski, François Morneau, Marcin Myszkowski, Kinga Nagy, Mats Nilsson, Thomas Nord-Larsen, Damjan Pantic, Jerôme Perin, John Redmond, Maria Rizzo, Vladimír Šebeň, Mitja Skudnik, Arnor Snorrason, Radosław Sroga, Todor Stoyanov, Arvid Svensson, Andrzej Talarczyk, Sander Teeuwen, Esther Thürig, José Uva, Sarah Mubareka

**Affiliations:** 1 European Commission, Joint Research Centre, Ispra, Italy; 2 Consultant to the European Commission, Joint Research Centre, Ispra, Italy; 3 Max Planck Institute for Biogeochemistry, Jena, Germany; 4 Forest Management Institute, Brandýs nad Labem-Stará Boleslav, Czech Republic; 5 Institute of Forest Science (INIA, CSIC), Crta. de la Coruña km 7.5, E-28040 Madrid, Spain; 6 Centro de Estudos Florestais, Instituto Superior de Agronomia, Universidade de Lisboa, Tapada da Ajuda, 1349-017 Lisboa, Portugal; 7 Thünen Institute of Forest Ecosystems, Alfred-Möller-Str. 1, 16225 Eberswalde, Germany; 8 University of Belgrade - Faculty of Forestry, Kneza Višeslava 1, 11 000 Belgrade, Serbia; 9 National Forest Centre, T.G. Masaryka 22, 96001 Zvolen, Slovakia; 10 University of Suceava, Faculty of Forestry, 13 University Street, Suceava, Romania; 11 IGN, ENSG, Laboratoire d’Inventaire Forestier (LIF), 14 rue Girardet, F-54000 Nancy, France; 12 Norwegian Institute of Bioeconomy Research (NIBIO), P.O. Box 115, NO-1431 Ås, Norway; 13 University of Zagreb - Faculty of Forestry and Wood Technology, Department of Forest Inventory and Management, Zagreb, Croatia; 14 Département d’analyse des forêts et des haies bocagères, Institut national de l’information géographique et forestière (IGN), 1 rue des Blanches Terres, 54250 Champigneulles, France; 15 Council for Agricultural Research and Economics, Research Centre for Forestry and Wood, Trento, Italy; 16 Latvian State Forest Research Institute “Silava”, 111 Rigas str., Salaspils, LV-2169 Latvia; 17 Swiss Federal Research Institute WSL, Zürcherstrasse 111, 8903 Birmensdorf, Switzerland; 18 Federal Research and Training Centre for Forests, Natural Hazards and Landscape (BFW), Seckendorff-Gudent-Weg 8, 1131 Vienna, Austria; 19 Department of Forest Resource Management, Swedish University of Agricultural Sciences, Umeå, Sweden; 20 Natural Resources Institute Finland (Luke), Latokartanonkaari 9, FI-00790 Helsinki, Finland; 21 Lithuanian State Forest Service, Pramonės av. 11A, LT-51327 Kaunas, Lithuania; 22 Københavns Universitet, Institut for Geovidenskab og Naturforvaltning, Rolighedsvej 23, 1958 Frederiksberg C, Denmark; 23 Université de Liège, Place du 20-Août 7, B-4000 Liège, Belgique; 24 National Institute for Research and Development in Forestry, 128, Eroilor Boulevard, Voluntari, Romania; 25 Bureau For Forest Management and Geodesy, ul. Leśników 21, 05-090 Sękocin Stary, Poland; 26 Hungarian National Land Centre, Forestry Department, Frankel Leó út 42-44, 1023 Budapest, Hungary; 27 Department of Agriculture, Food and the Marine, Johnstown Castle Estate, Wexford, Y35 PN52 Ireland; 28 Slovenian Forestry Institute, Department for Forest and Landscape Planning and Monitoring, Ljubljana, Slovenia; 29 Biotechnical Faculty, Department of Forestry and Renewable Forest Resources, University of Ljubljana, Ljubljana, Slovenia; 30 Icelandic Forest Research, 162 Reykjavik, Iceland; 31 Forest Research Institute, Bulgarian Academy of Sciences, 132, “St. Kliment Ohridski” Blvd., 1756 Sofia, Bulgaria; 32 Forest and Natural Resources Research Centre Foundation/Taxus IT, ul. Płomyka 56A, 02-491 Warsaw, Poland; 33 Stichting Probos, 6700 Wageningen, AG The Netherlands; 34 Institute for Nature Conservation and Forests, Av. da República 16, 1050-191 Lisboa, Portugal

**Keywords:** Forest ecology, Carbon cycle, Forestry

## Abstract

Forest biomass is an essential resource in relation to the green transition and its assessment is key for the sustainable management of forest resources. Here, we present a forest biomass dataset for Europe based on the best available inventory and satellite data, with a higher level of harmonisation and spatial resolution than other existing data. This database provides statistics and maps of the forest area, biomass stock and their share available for wood supply in the year 2020, and statistics on gross and net volume increment in 2010–2020, for 38 European countries. The statistics of most countries are available at a sub-national scale and are derived from National Forest Inventory data, harmonised using common reference definitions and estimation methodology, and updated to a common year using a modelling approach. For those counties without harmonised statistics, data were derived from the State of Europe’s Forest 2020 Report at the national scale. The maps are coherent with the statistics and depict the spatial distribution of the forest variables at 100 m resolution.

## Background & Summary

Forest biomass is becoming increasingly important for several forest-related policies in the European Union (EU) under the Green Deal^1^. However, biomass is a finite renewable resource. Increased demand for forest biomass related to the green transition and climate neutrality raises questions regarding the biomass availability to satisfy this demand^2–4^. Thus, an accurate and updated assessment of the available forest biomass stock and related growth rate is essential for the formulation of scientifically robust and coherent forest policies and management, and better balance the different and sometimes competitive interactions between their ecosystem services^5–7^.

Most European countries have implemented a sample-based National Forest Inventory (NFI) to regularly obtain reliable statistics on forest biomass resources with a revisit frequency that typically ranges between 5 and 10 years^8^. Owing to their mission to provide the best possible estimates for decision-making within a country, NFIs typically employ country-specific definitions and inventory designs that substantially differ from each other (e.g.^8,9^). While more and more NFIs provide data with fine-scale spatial resolution (e.g.^10–14^), most countries deliver area-level statistics at national or subnational scale. Consequently, at the European level, NFI data refer to different periods, biomass pools, and spatial scales that hamper their comparability and integration^9,15^.

The use of different definitions may cause inconsistencies when integrating biomass data at international or global scale, or when comparing countries’ inventories with model results^16,17^. Moreover, spatial disaggregation of national forest inventory data to finer scales, enabling the wall-to-wall mapping of forest resources, would be essential for a variety of applications, such as a better assessment of biomass accessibility and extraction costs and their impacts on the local socio-economic forestry system^9,18,19^.

Here, we present a reference dataset that provides a comprehensive and detailed view of the current biomass resources in Europe. It contributes to a better assessment of the status of European forests and their capacity to provide biomass and other ecosystem services. Our dataset provides harmonised statistics on standing forest biomass and its share available for wood supply, accompanied by consistent 100 m (1 ha) spatial resolution maps, and harmonised statistics on volume increment of European forests. Unlike internationally-reported statistics providing values at national scale (e.g., Forest Europe, FAO, ESTAT) or satellite-based maps that often present large discrepancies with NFI statistics, we provide values at sub-national scale for most countries along with maps that are consistent with the NFI data.

This dataset is the result of a multi-annual dedicated effort and collaboration of several European NFIs under the coordination of the European National Forest Inventory Network (ENFIN) with the Joint Research Centre (JRC) of the European Commission for an ad-hoc harmonisation of the NFI biomass data. In this study, the NFI institutions initiated the process by harmonising their national statistics using a common reference definition and methodology for the estimation^20^. In a second step, the JRC temporally aligned the biomass data to a common year either by using the Carbon Budget Model (CBM), a forest growth model calibrated for each country using NFI data^21^, or by applying linear adjustment functions. For the countries that could not be included in this harmonisation process, the reference statistics were derived from the State of Europe’s Forests (SoEF) 2020 database^22^ at national scale.

In order to produce a complete reference dataset and support multiple uses, the missing values in the SoEF database were predicted using approximations from the existing data, either from the same country or neighbouring countries. Depending on the data source and method used to obtain the forest variables, an uncertainty quality indicator was assigned to each estimate. The uncertainty value is equal to 0 for harmonised NFI data (lowest uncertainty), 1 for values derived from the SoEF database, 2 for missing values predicted using data from the same country, and 3 for missing values extrapolated using data from other countries (highest uncertainty). The data source and spatial scale (national or subnational) of the statistics for each forest variable and country are reported in Table 1.

**Table 1:** List of the 38 European countries included in this dataset, with their ISO2 code, the country name, the level of spatial resolution of the statistics according to the NUTS classification, the data source of each forest variable, and the reference year and period of the NFI data used in this study (before temporal harmonisation).

Country		NUTS level		DATA SOURCE					NFI cycle	
ISO	Name	Biomass, FAWS	Increment	Forest area	FAWS	Biomass	BAWS	Increment	Ref. year	Start-End
AD	Andorra	0	0	SoEF	GF	GF	GF	GF		
AL	Albania	0	0	SoEF	SoEF	SoEF	SoEF	SoEF (gf2)		
AT	Austria	2	2	NFI & SoEF	NFI	NFI	NFI	NFI (gf1)	2008	2007––2009
BA	Bosnia and Herzegovina	0	0	SoEF	GF	GF	GF	GF		
BE	Belgium	1	0	NFI & SoEF	SoEF	NFI	SoEF	SoEF	2012	2008–2015
BG	Bulgaria	2	0	NFI & SoEF	NFI	NFI	NFI	SoEF (gf2)	2007	2001–2014
CH	Switzerland	2	0	NFI	NFI	NFI	NFI	SoEF	2005	2004–2006
CY	Cyprus	0	0	SoEF	SoEF	SoEF	SoEF	SoEF (gf1)		
CZ	Czech Republic	3	3	NFI	NFI	NFI	NFI	NFI (gf1)	2003	2001–2004
DE	Germany	2	2	NFI	NFI	NFI	NFI	NFI (gf1)	2002	2002
DK	Denmark	0	0	NFI & SoEF	SoEF	NFI	SoEF	SoEF	2014	2010–2014
EE	Estonia	0	0	SoEF	SoEF	SoEF	SoEF	SoEF		
ES	Spain	3	3	NFI & SoEF	NFI	NFI	NFI	NFI (gf1)	2002	1997–2007
FI	Finland	1	1	NFI & SoEF	SoEF	NFI	SoEF	NFI (gf1)	2006	2004–2008
FR	France	2	2	NFI & SoEF	NFI	NFI	NFI	NFI (gf1)	2010	2008–2012
GB	Great Britain	0	0	SoEF	SoEF	SoEF	SoEF	CBM		
GR	Greece	0	0	NFI & SoEF	SoEF	GF	SoEF	CBM (gf1)		
HR	Croatia	2	0	NFI	SoEF	NFI	SoEF	SoEF	2008	2006–2009
HU	Hungary	3	0	NFI	NFI	NFI	NFI	SoEF	2012	2010–2014
IE	Ireland	1	0	NFI	NFI	NFI	NFI	SoEF	2006	2006
IS	Iceland	2	0	NFI	NFI	NFI	NFI	SoEF (gf2)	2010	2005–2014
IT	Italy	2	2	NFI & SoEF	SoEF	NFI	SoEF	NFI (gf1)	2005	2003–2006
LI	Liechtenstein	0	0	SoEF	SoEF	SoEF	SoEF	SoEF (gf2)		
LT	Lithuania	3	0	NFI & SoEF	NFI	NFI	NFI	SoEF	2010	2008–2012
LU	Luxembourg	0	0	SoEF	SoEF	SoEF	GF	SoEF (gf2)		
LV	Latvia	0	0	NFI & SoEF	NFI	NFI	NFI	SoEF (gf1)	2011	2009–2013
ME	Montenegro	0	0	SoEF	SoEF	SoEF	SoEF	SoEF (gf1)		
MK	Macedonia	0	0	SoEF	SoEF	SoEF	SoEF	SoEF (gf2)		
MT	Malta	0	0	SoEF	GF	GF	GF	GF		
NL	Netherlands	2	0	NFI & SoEF	NFI	NFI	NFI	SoEF	2012	2012–2013
NO	Norway	3	0	NFI & SoEF	NFI	NFI	NFI	SoEF	2010	2008–2012
PL	Poland	2	2	NFI & SoEF	NFI	NFI	NFI	NFI (gf1)	2012	2010–2014
PT	Portugal	2	0	NFI	NFI	NFI	NFI	CBM (gf1)	2015	2015
RO	Romania	1	2	NFI & SoEF	NFI	NFI	NFI	NFI (gf1)	2011	2008–2013
RS	Serbia	2	0	NFI	NFI	NFI	SoEF	SoEF (gf2)	2005	2004–2006
SE	Sweden	2	2	NFI	NFI	NFI	NFI	NFI (gf1)	2011	2009–2013
SI	Slovenia	1	0	NFI	NFI	NFI	NFI	SoEF (gf1)	2012	2012
SK	Slovakia	3	0	NFI	NFI	NFI	NFI	SoEF	2006	2005–2006

**NUTS level**: hierarchical system to divide the territory from national level (NUTS 0) to small regions (NUTS 3). **SoEF**: Data derived from SoEF 2020 database, available only at national level. The SoEF data have been modified with gap-filling procedures compared to the original values only where indicated. **NFI**: Data derived from the harmonised NFI data, available at sub-national level, updated using SoEF data but not matching the SoEF values. The NFI data used in this study have been always processed, updated and harmonised, and thus differ from the NFI data provided by the original data source. **CBM**: Data derived from the Carbon Budget Model (CBM). **SoEF & NFI**: Data derived from the harmonised NFI data at sub-national level, updated to match the SoEF values at national level. When the data source did not provide all variables, the missing values were estimated using gap-filling procedures. **GF**: Data completely derived using gap-filling procedures, using data from other countries. **gf1**: Data partly derived from gap-filling procedures, using data from the same country. **gf2**: Data partly derived from gap-filling procedures, using data from other countries.

The JRC used the harmonised statistics as calibration data to produce maps of forest area, forest area available for wood supply, biomass stock, and biomass stock available for wood supply consistent with official statistics. These maps were either produced on purpose or obtained by adjusting existing products. Compared to the statistics, the maps have the added value of describing the fine-scale variability of the forest variables and, considering their uncertainty, can be used for multiple management and modelling purposes, from quantifying the harvesting cost to supporting the estimation of the carbon fluxes from the forest sector^23,24^.

**Fig. 1:**
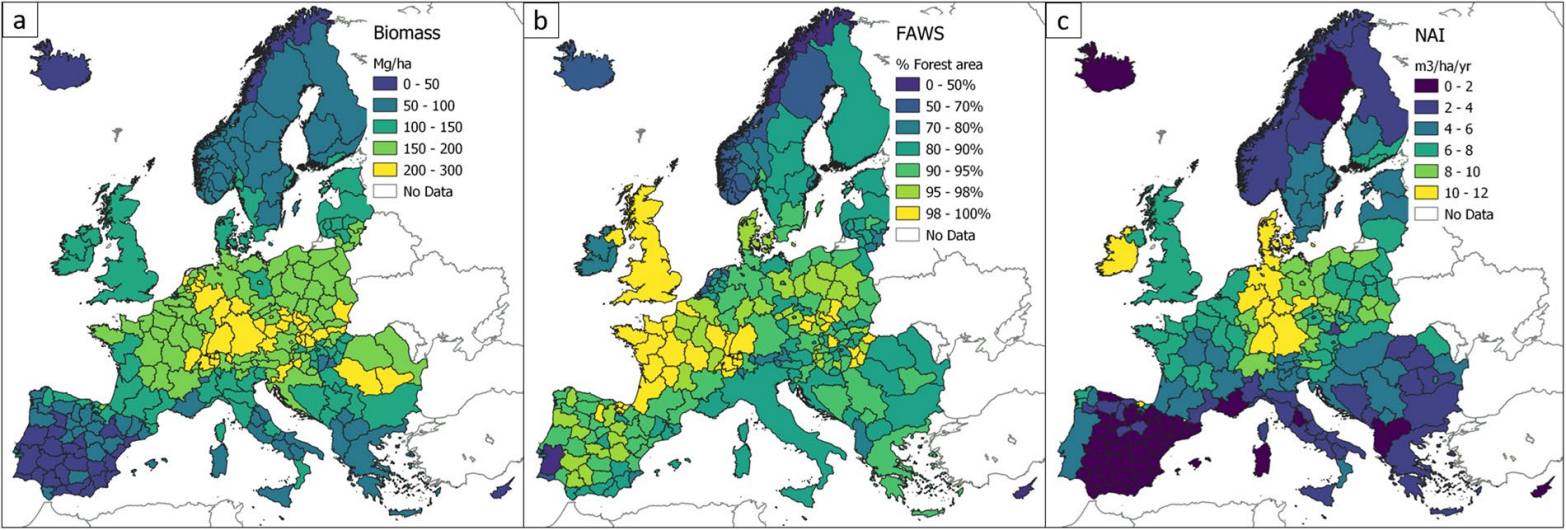
Reference forest statistics at administrative level for Europe. (**a**) mean forest AGB/ha (Biomass); (**b**) percent of forest area available for wood supply (FAWS) with respect to the total forest area of the administrative unit; (**c**) net annual increment in forest areas (NAI). The spatial resolution (national or sub-national) of the statistics varies according to the forest variable and country, reflecting the spatial detail provided by the data source.

**Fig. 2:**
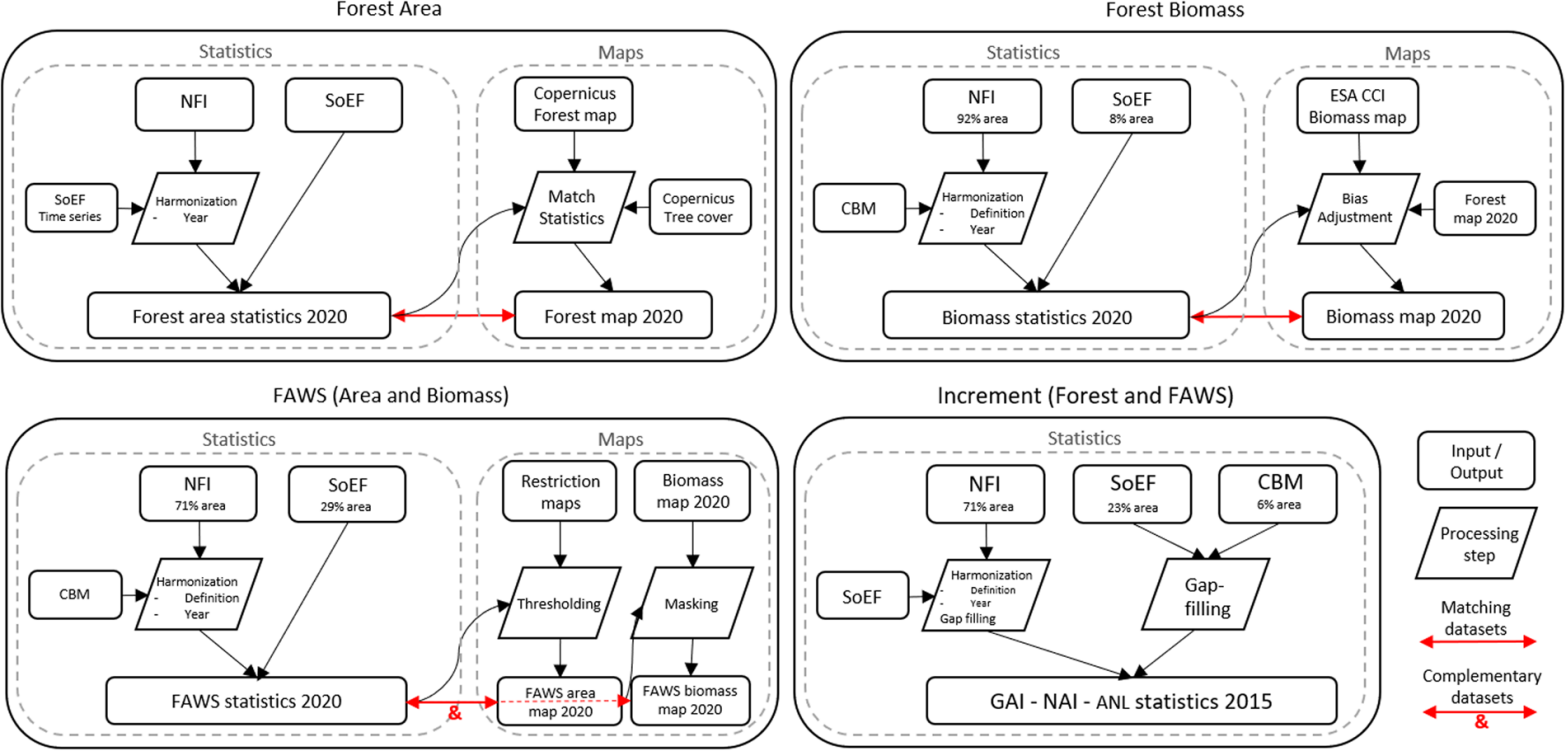
Flowchart of input and output data and processing steps. The NFI data are available at national and sub-national scales, while the SoEF and CBM data are available only at national scale, and their percentages refer to the forest area of Europe represented by each dataset.

Our reference dataset includes 38 European countries; its spatial coverage and resolution are shown in Fig. 1. Figure 2 summarises the input and output data, along with the necessary processing steps. Due to the harmonisation procedure, this dataset may differ from the official data reported by the NFIs in terms of their national and international reporting of forest area, biomass stock and increment. We acknowledge that national definitions are designed to best represent the specific conditions of the country’s forests^8^. Using a common definition or approach in several diverse countries may not fit their forest characteristics as well as the national definitions do. Thus, the NFI data based on national definitions and methodologies remains the main data source for within-country assessments. On the other hand, harmonisation is essential to perform assessment of forest resources, calibrate large-scale models or design policies at European scale in a coherent way. Thus, the added value of this Europe-wide set of statistics and maps lies in their higher level of harmonisation, completeness, consistency and spatial resolution compared to other existing statistics (e.g.^22^) and maps^25^. The components of our reference dataset are described in the following sections.

### Forest biomass: statistics

The biomass statistics refer to the aboveground standing biomass of all living trees, including the aboveground stump, the stem from stump to top, branches and foliage (AGB) as total AGB stock (tons) and AGB stock per hectare (AGB/ha) (t/ha) in the forest areas of each country. The biomass statistics of 26 countries (see country list in the Methods section), representing 92% of the forest area of Europe^22^, were obtained by harmonising the national data in terms of the biomass definition and temporal resolution (Fig. 1a). Specifically, the NFI biomass data were first of all adjusted at tree level with ad-hoc correction and expansion factors to include the same components of the trees, namely all aboveground parts from stump to top (included), with dead and living branches and foliage^26–29^. The harmonised statistics were then updated to the year 2020 using the CBM, a modelling approach that simulates the biomass changes due to forest growth, mortality, harvest and natural disturbances^21^. Biomass changes due to afforestation and deforestation were not modelled by CBM. They were estimated according to the average biomass density of young forests (<20 years) (for afforestation) and of all forests (for deforestation).

For most countries, the biomass stock refers to the forest area reported in the SoEF, unless differences between the area reported by the NFI and the SoEF are substantial (see Methods). The harmonised biomass statistics are available at sub-national level based on the Nomenclature of Territorial Units for Statistics (NUTS), varying by country from NUTS-1 to NUTS-3. For the remaining countries, representing 8% of the forest area of Europe, the biomass stock for 2020 is derived from the SoEF database and is therefore available only at national scale (Fig. 1a).

### Forest biomass: map

The existing forest biomass maps for Europe show substantial differences from the reference data, which can be larger than 50% at the pixel level^25,30^. In this study, the harmonised biomass statistics were used to correct the systematic bias of a published biomass map^31^ at the spatial scale of the statistics. In order to properly match the AGB per hectare, the map and the statistics must refer to the same forest area. Thus, we first produced a forest mask that matched the forest area statistics, and applied it to the biomass map. For this task, we modified the Copernicus Forest Type map for 2018^32^ to obtain a forest mask coherent with the statistics. Then, we applied this forest mask to the European Space Agency’s (ESA’s) Climate Change Initiative (CCI) biomass map for 2020^31^, which was selected because it showed good accuracy for Europe^30^. Then, the biomass map was bias-adjusted to match the reference biomass stock statistics at national and sub-national levels. The resulting biomass map depicts the AGB per hectare of European forests at 100 m resolution, which is consistent with our harmonised statistics for the year 2020.

### Forest available for wood supply: statistics

Updated and comparable statistics on the area and biomass stock of the Forest Available for Wood Supply (FAWS) in Europe are essential to better assess the current biomass resources available for the bioeconomy, to understand the factors limiting wood availability, and to model the potential wood supply in the future. As for the biomass statistics, the NFI data from 20 countries (representing 71% of the forest area of Europe) on forest area and biomass (AGB) available for wood supply at sub-national scale were first harmonised using a common definition and methodology^33^ and then updated to the reference year 2020 (Fig. 1b). Forests are considered as FAWS where restrictions do not have a significant impact on the current or potential wood supply^34^. The common definition identifies a set of restrictions due to environmental, social or economic limitations. The harmonisation procedure allowed us also to quantify the impact of each restriction on reducing the forest availability at sub-national level, which is essential to support and guide the mapping of FAWS using remote sensing data, or to model the availability of wood resources at a fine spatial scale. For the countries not included in the harmonisation study, we derived the FAWS statistics from the SoEF database at national scale.

### Forest available for wood supply: map

The FAWS was mapped in terms of area and biomass (AGB/ha). The harmonised FAWS area statistics for each restriction were used to map the main limitations to wood availability. These are forest areas not available due to steep slope, high altitude, protected areas, protected species, poor accessibility, and low productivity. The FAWS area map was obtained by removing areas not available for wood supply from the forest area map matching the statistics. Then, the FAWS biomass map was obtained by masking the bias-adjusted biomass map described above with the FAWS area map.

The FAWS maps use consistent forest area and biomass definitions and approaches resulting in coherent maps across Europe. Still, they are not designed to match the FAWS area and biomass statistics because maps and statistics do not always identify the same restrictions. Therefore, the FAWS maps are complementary to the FAWS statistics and their integration can provide a more complete assessment of the FAWS area and biomass than each of them independently. In fact, some restrictions on wood availability estimated from plot data and thus included in the FAWS statistics (e.g., social or protective functions of the forests) could not be mapped due to the lack of spatial data related to these restrictions. Conversely, the FAWS statistics of some countries did not include some mapped restrictions due to a lack of ground information. In several countries, the two datasets match well because some restrictions that could not be mapped occur in the same area as others that were mapped. For example, forests protected to prevent erosion (not mapped) are usually located on steep slopes (mapped). However, the FAWS maps can certainly be refined at national scale using country-specific and more detailed maps of restrictions. In contrast, the FAWS statistics of some countries can be integrated with the information provided by the maps to assess the restrictions not included in the ground protocol.

### Forest increment: statistics

Forest management and international reporting require the assessment of the forest growth to estimate the amount of wood that can be sustainably harvested and the carbon sequestration due to the net biomass accumulation in the forest. Here, we report the Gross Annual Increment (GAI), the Net Annual Increment (NAI) and the Annual Natural Loss (ANL) of European forests, where GAI = NAI + ANL. Our dataset refers to the volume increment per year (m^3^/year) and provides consistent data for the GAI, NAI, and ANL for the forest and FAWS area in Europe for 2015. Since the increment is computed from two separate measurements in time, the year 2015 represents the reference year of a reporting period ranging 2010–2020 for the NFI data and 2013–2017 for the SoEF data. When the NFI data referred to a different time period, they were temporally adjusted using correction factors.

The increment statistics are harmonised for the definition, estimation method and reference year at sub-national scale for 10 countries, representing 71% of the European forest area (Fig. 1c). For the remaining countries, the increment data are obtained at national scale from the SoEF database for 25 countries (23% of the European forest area) and by using the CBM model runs for 3 countries (6% of the European forest area) that did not report increment values in the SoEF. For all countries, the increment data refer to the forest and FAWS area reported by the SoEF for 2015. Since not all countries provide GAI and NAI for forest and FAWS area, the missing values were estimated using linear adjustments from the available data.

## Methods

The dataset contains harmonised statistics and maps for the European forests regarding the forest area, the forest biomass stock, the area and biomass available for wood supply, and the forest increment, which includes the growth (gross and net increment) and the losses due to natural causes. Most results were derived by harmonising national statistics and published maps using common definitions and estimation methods. The data were further updated to the reference year 2020 for the biomass stock and the biomass available for wood supply, and the year 2015 for the forest increment. Each product is described in detail in the following sections. Throughout the text, we note that the NFI data refer to the data produced for this study using the harmonised definitions and approaches, and not to the data produced for national reporting. Moreover, since the production of the harmonised dataset used here, the NFI may have released more recent data using national definitions and approaches.

### Forest area: statistics

The statistics of this dataset refer, for most countries, to the forest area reported in the SoEF database. In fact, most NFIs provided harmonised biomass and increment statistics that refer to the FAO forest definition^35^, which is used in the SoEF database, and thus the NFI forest area in the reporting year and the (closest in time) SoEF value were comparable. The difference in forest areas was considered negligible when it was smaller than 2%, likely due to approximations because the NFI data are attributed to the NFI reporting year but are usually acquired during a longer period. In such cases, when the NFI statistics were updated to a common reference year, their forest area was matched to the SoEF value, and the forest area of the sub-national administrative units was linearly adjusted so that their sum corresponded to the national value.

Regarding forest volume increment, the harmonised statistics refer to the forest area reported in the SoEF for all countries. Instead, in the case of forest biomass and FAWS, the data provided by the NFIs of 8 countries (CH, CZ, DE, HR, HU, IS, RS, SK), representing 13% of the European forest area, referred to the national forest definition (or a forest definition applied specifically to produce the harmonised biomass data). Their forest area differed more than 2% from the SoEF values, with 5 countries (DE, HR, IS, RS, SK) presenting an area difference >5%. In such cases, we estimated their forest area in the reference year according to the NFI forest definition. This value was obtained by adding to the NFI forest area in their reporting year the area change provided by the SoEF time series. Thus, for these 8 countries we report the biomass stock for a forest area in 2020 that differs from the value used in the SoEF, and it is not directly comparable with the increment statistics, which refer to the SoEF forest area for all countries. For example, the NFI of CZ reports for 2003 a forest area of 2.75 million ha (Mha), while the forest area in SoEF in 2000 and 2005 is 4% lower (2.64 and 2.65 Mha, respectively). Considering that CZ reports in the SoEF an increase of forest area of 2,000 ha (0.1%) per year between 2000 and 2020, this annual area change was added to the NFI forest area in 2003, multiplied for the years (17) between the NFI reporting year (2003) and the year 2020.

The differences in forest area between the NFI data and the SoEF database for these 8 countries were due to various reasons. In the case of CH, the NFI data exclude the inaccessible forest areas due to the lack of ground data, which were included in the SoEF using extrapolations from the data acquired in accessible forests. In CZ, the NFI data include the areas with dwarf mountain pine (*Pinus mugo* T.) stands, classified as Other Wooded Land (OWL) and Other Land with Tree Cover and thus excluded in the SoEF. In DE, the NFI data exclude unstocked forest areas and inaccessible areas, included in the SoEF. In HR, the NFI data include the area classified as OWL (considered unproductive forest), excluded in the SoEF. In HU, the NFI uses 10% as the minimum tree cover, while the SoEF forest area refers to 30%. In IS, the NFI and SoEF forest area are estimated using different methods: the NFI uses plots acquired during a long period (2005–2014), while the SoEF forest area is obtained using extrapolation and regression of previous estimates. In RS, the NFI data include the area classified as OWL, partly excluded in the SoEF. In SK, the SoEF data refer only to the forest area recorded in the cadastre as forest land, while the NFI data include all forests, also those not recorded in the cadastre.

### Forest area: map

A forest area map matching the reference statistics was produced to support the mapping of the forest biomass stock and its share available for wood supply. The forest area map was obtained by modifying the Copernicus Forest Type 2018 map at 100 m^32^. The Copernicus map was selected because it is compatible with the spatial and temporal resolutions of the biomass maps and the FAO forest definition used by most reference biomass statistics as it employs a minimum mapping unit of 0.5 ha, a minimum tree cover density of 10%, and excludes the trees under agricultural use and urban context.

The Copernicus Forest Type map was first converted to a forest and non-forest mask aggregating all the forest types in one class. Then, this forest mask was adjusted to match the forest area reported by our statistics at sub-national scale using, as additional information, the Copernicus 2018 Tree Cover Density map^32^ as follows. When the statistics reported a smaller forest area than the forest mask, the forest areas in the mask with lower tree cover were converted to non-forest, until the mask matched the statistics. Alternatively, when the statistics reported a larger forest area than the forest mask, the areas in the mask with higher tree cover but located outside forest and outside the areas with tree cover in urban and agricultural contexts (as identified by the Copernicus support layers) were converted to forest. Usually, the forest mask was expanded around the forest edges with high tree cover that were not included in the forest map because of edge effects (geolocation mismatches), and then in forest areas with lower tree cover. The resulting forest mask matches the forest area reported by the reference statistics at sub-national scale.

### Forest biomass: statistics

The statistics of the forest biomass are provided in terms of total AGB (tons) and AGB per hectare (t/ha) and refer to the year 2020. These values are produced by compiling, processing and harmonising the available NFI data at sub-national level or, in their absence, by using the statistics reported in the SoEF 2020 database at national scale^22^. The statistics are fully harmonised for aboveground biomass (i.e., the data refer to the same components of the trees) and reference year (i.e., the data refer to the same year, 2020) for 26 countries representing 92% of the European forests (AT, BE, BG, CH, CZ, DE, DK, ES, FI, FR, HR, HU, IE, IT, IS, LT, LV, NL, NO, PL, PT, RO, RS, SE, SI, SK), as described in details below. These statistics are available at sub-national level for 24 countries and at national scale for two countries (IS, LV). The sub-national administrative units used in this study were harmonised to the 2016 Nomenclature of Territorial Units for Statistics (NUTS) classification. For the remaining 8% of the European forests (AD, AL, BA, CY, EE, GB, GR, LI, LU, ME, MK, and MT), the biomass statistics were derived from the SoEF database. In the case of GR, since the SoEF data were obtained using linear extrapolation of old inventory data (i.e., the NFIs of 1964 and 1992), the SoEF biomass statistics for 2020 were modified to adjust them with the values reported by neighbouring countries (see Supplementary Information). The SoEF reports aboveground biomass in units of carbon stock converted to biomass using the carbon fraction for dry biomass used by the country in the SoEF database or, if not reported, the default value of 0.5, which is used by most countries.

We note that, even though the SoEF database provides time series of forest statistics at national scale for the period 1990–2020, the NFI statistics harmonised for biomass definition and reference year were preferred, when available, for two reasons. Firstly, the harmonised NFI statistics are available at sub-national level, describing the spatial distribution of the biomass stocks within the country, compared to the summary values at national scale provided by the SoEF. Secondly, the SoEF data have a lower level of comparability because the harmonisation of definitions and reference year usually is not based on data modelling. Instead, the SoEF harmonisation is often performed with a linear extrapolation of the NFI statistics or using expected values based on expert knowledge (e.g., in national forecasts or outlook studies), or it is not performed, and the closest available NFI values are used^22,36^.

#### Harmonisation of biomass pool

The forest biomass data produced by the NFIs for national reporting are not directly comparable because they employ different definitions regarding the biomass pool, namely what parts of the tree are included and the minimum diameter threshold applied. In addition, NFIs may employ different approaches to estimate the biomass from the tree parameters (i.e., country-specific allometric equations or biomass conversion and expansion factors), which influence the resulting values. For these reasons, several European NFI institutions, under the coordination of the ENFIN cooperated to address the differences indicated above and to achieve a better harmonisation of the forest biomass statistics in Europe. The NFIs identified a harmonised biomass definition and a common estimator, which were applied to the NFI plot-level data to obtain biomass estimates at the administrative level referring to the same biomass pool and estimation method for all countries^28,29^.

The harmonised biomass definition includes all aboveground biomass compartments of the living trees, namely the aboveground part of the stump, the stem from stump to top, dead and living branches, and foliage. This definition is comparable to the definition used in the SoEF for reporting carbon in aboveground biomass, which includes all living biomass above the soil, including stems, stumps, branches, bark, seeds, and foliage^35^. The common estimator, called e-Forest, is a design-based unbiased estimator applicable to any plot data regardless of the original NFI sampling design^37^. The common definition and estimator were applied to 431,261 field plots to obtain estimates harmonised for biomass pool at national and sub-national levels for 26 European countries^28,29^. The forest biomass estimated using the harmonised definition is usually higher compared to the value based on the national definitions because the latter often does not include all aboveground biomass compartments, such as leaves or aboveground parts of the stump. The difference in total biomass between harmonised and national definitions was found to be 4% for the 26 countries^25^.

#### Harmonisation of reference year

Each NFI acquires field data during different time periods that are not synchronised across countries. Consequently, the biomass statistics harmonised for the biomass pool described above are not temporally harmonised, but are derived from plot data ranging from 2001 to 2013. However, the biomass stock may change substantially in a period of almost 20 years because of forest growth, mortality, harvest and changes in forest area (deforestation and afforestation). Given the need for coherent and updated statistics, we further harmonised the biomass statistics to a common reference year (i.e., 2020, at the end of the calendar year) using the Carbon Budget Model (CBM). The CBM is an inventory-based, yield-curve-driven model that simulates the stand- and landscape-level carbon dynamics of all forest carbon pools using information on age structure, management practices, harvest regimes, and natural disturbances^38^. The model, developed by the Canadian Forest Service, was adapted to the specific European conditions and applied to EU countries to estimate the forest carbon dynamics at national and sub-national level^39–42^.

In this study, we used an updated version of the CBM model, named JRC Forest Carbon Model, which was recently developed by the JRC in collaboration with the Canadian Forest Service^39^. For each EU Member State, all the main input data used by the model were preliminarily updated and validated against independent data sources^43^. The model was used to quantify the biomass density at national level for the NFI year and the reference year 2020 for the EU countries. Then, the difference between the CBM biomass density for 2020 and the NFI year, divided by the value in the NFI year, provided the percentage biomass net change (gain or loss), which was applied as a correction factor to the NFI statistics to update them to the reference year. This correction considered only the biomass change due to natural growth, mortality and harvest on the forest area reported by the NFI. The biomass changes due to the variations in the forest area (i.e., net afforestation or deforestation) were computed as follows. The biomass gains due to net afforestation were estimated using the growth rate for young forests (i.e., the biomass density of forest stands in the age class 0–19 years) computed by the CBM at sub-national scale, while the biomass losses due to net deforestation were quantified using the average forest biomass density of the sub-national administrative unit. Currently, the CBM is not parameterized for all the 26 countries with statistics harmonised for biomass pool. For 7 countries (CH, ES, IE, IS, LV, NO, RS), the percentage biomass change was derived from the SoEF time series of aboveground biomass stock. For other 4 countries (CH, IS, NO, RS) the growth rate for young forests was derived from neighbouring administrative units included in the CBM.

Thus, the harmonised statistics on the biomass stock of the 26 countries were updated to the year 2020 considering the biomass stock change on stable forest land due to natural growth and forest management, and the biomass growth due to net forest expansion or the biomass loss due to net forest loss. This approach was also used to update the statistics of the biomass available for wood supply. The net change in the biomass stocks due to the forest dynamics ranged from 0.1% to 1.8% per year at national level (besides IE and IS, where it was higher due to the large afforestation areas with fast-growing trees). When considering the total change between the NFI year (variable by country) and the reference year 2020, the biomass stock of the 26 countries increased overall by 11.3%, showing the relevance of the temporal harmonisation to update the NFI statistics, especially for the countries where the latest NFI was completed several years ago. Most of the biomass increase (10.7%) was due to the forest growth on stable forest land, while only 0.62% of the increase was due to the net biomass change in afforestation (+0.67%) and deforestation (−0.05%) areas.

### Forest biomass: map

The error of a map (or, its difference compared to a reference dataset) can be distinguished in two components: the systematic error (or, bias) and the random error of pixel-level predictions. In the case of biomass maps, the bias is often due to calibration data with systematic errors in their estimates or extrapolation issues^44^, inaccurate model parameters and the limited sensitivity of the remote sensing data to biomass variability. Several studies have reported that forest biomass maps tend to overestimate the stock in areas with low biomass density and underestimate the stock in areas with high biomass density, thus they are affected by different systematic errors at different biomass ranges^25,45,46^. Instead, random errors are mostly related to the noise inherent in the satellite data and to the uncertainty in the algorithms for biomass retrieval. While random errors are unavoidable but can be minimised by spatial or temporal averaging, systematic errors can be corrected (bias-adjustment) using reference data obtained from a statistical sample and an unbiased estimator^47^.

We modified the ESA CCI biomass map for 2020^31^ to remove the difference at sub-national scale with the harmonised biomass statistics for 2020 described above. In order to match the map with the reference statistics both in terms of forest area and AGB/ha, we first applied a forest mask consistent with the forest area statistics (adjustment of forest area). Then, we estimated the AGB/ha in the areas with tree cover but no biomass values in the map (adjustment of areas with no biomass), and corrected the map to ensure its compatibility with the statistics at sub-national scale (adjustment of systematic difference). We note that this approach corrects systematic differences with the reference data at administrative level, but the random errors remain and affect the map accuracy at local and pixel levels.

#### Adjustment of forest area

The biomass statistics refer to a certain forest area. A systematic difference between the statistics and a map can occur because they refer to different areas, either because they employ a different forest definition or different methods to quantify it. For example, the reference statistics may have lower biomass density than the biomass map in the administrative units where they represent a larger forest area because they include sparse forests with low biomass. Conversely, the statistics may have higher biomass density than the map in the administrative units where they represent a smaller forest area because they refer to denser and high-biomass forests. Hence, the bias correction of a biomass map using reference statistics should first match their forest area. Here, the ESA CCI biomass map, provided without a forest mask, was masked using the forest area map described above, which matches the forest area reported by the reference statistics at sub-national scale.

#### Adjustment of areas with no biomass

After removing the non-forest areas, the ESA CCI biomass map presented zones in the Iberian Peninsula with no biomass while the Copernicus Forest Type and Tree Cover maps indicate the presence of forest with substantial tree cover (up to 50%). When applying a bias-correction approach, the presence of large forest areas with zero biomass would be compensated by higher correction factors, leading to biomass overestimation at the local scale in the output map. To avoid such artefacts, the biomass density in the areas of the map with zero values but the presence of trees was estimated using the local relation between biomass and tree cover density. Tree cover is spatially correlated with biomass density, and it has been used as biomass predictor at national^48^ and international level^49^. This relation varies with the forest type and saturates with canopy closure. However, it may be relatively strong during the initial phases of forest development and when calibrated at local scale. In this study, the relation between tree cover and biomass was estimated specifically for the central-southern Iberian Peninsula (part of ES and PT) from the Copernicus and CCI maps using linear models for the forest areas, excluding the areas with trees outside forest. This relation was then applied to correct the biomass map in the forest areas with zero biomass and located in the same region where the model was calibrated.

#### Adjustment of systematic difference

The ESA CCI biomass map, modified as indicated above, was then corrected by removing the systematic difference (i.e., the bias) with respect to the biomass density reported by our reference statistics at sub-national scale. The bias was removed using a correction factor, computed as the ratio between the biomass density of the reference statistics and the mean value of the biomass map over the same area represented by the reference statistics^46^. The correction factor was computed at the scale of the administrative units of the reference data, and then applied to the biomass map at pixel level. Thus, the resulting bias-adjusted biomass map matches the reference statistics at sub-national scale. Compared to the original map, the adjusted map presents lower biomass density in Finland and the Baltic states, in Eastern Germany, in the northern part of the Iberian Peninsula and in southern Greece; higher biomass density in the central and southern parts of the Iberian Peninsula, in western Europe (France, the Netherlands, UK and Ireland) and in the western Balkans; comparable biomass density in central and Eastern Europe and in Scandinavia.

We note that the bias could have also been estimated at country level using a linear or non-linear modelling approach^47^, such as a regression between the mean biomass values of the statistics and of the map at sub-national scale. However, in the context of this study, the model parameters would have substantial uncertainty because the bias of the map varies according to complex environmental factors rather than the administrative units. Moreover, most countries present a small number of sub-national units (<10) from which to estimate the parameters. Such sources of uncertainty of the model parameters would likely cause a mismatch between the bias-adjusted map and the reference statistics at sub-national scale.

### Forest available for wood supply: statistics

The FAWS statistics are provided in terms of FAWS area (ha) and FAWS biomass (t/ha), and refer to the year 2020. The FAWS statistics are harmonised for reference definition and reference year for 20 countries representing 71% of the European forest area (AT, BG, CH, CZ, DE, ES, FR (only FAWS area), HU, IE, IS, LT, LV, NL, NO, PL, PT, RO, SE, SK, SI). The FAWS area and biomass statistics were obtained from the SoEF database at national scale for the remaining countries, if reported. Since the SoEF reports the wood availability only in units of Growing Stock Volume (GSV), the biomass available for wood supply was derived by multiplying the total biomass stock with the ratio between the GSV available for wood supply and the total GSV. Four countries (AD, BA, MT, RS), corresponding to 3% of the area and 2% of the biomass stock of European forests, did not report on the share of area or biomass available for wood supply in the SoEF and these values were estimated using data from neighbouring countries (see Supplementary Information).

As for the biomass stock, the harmonised FAWS statistics, when available, were generally preferred to the SoEF data for three reasons. Firstly, the SoEF database provides time-series of FAWS statistics for the period 1990–2020 in terms of area and growing stock, but it does not report information on the biomass or carbon stock available for wood supply. Secondly, the harmonisation of definitions and reference year of the SoEF data is highly variable according to the country, and it is usually performed either with a linear extrapolation of the NFI data, or using expected values based on expert knowledge, or simply using the closest available NFI values. Thirdly, the statistics provided by the SoEF are published only at national scale, while the harmonised FAWS data are available at sub-national level.

#### Harmonisation of FAWS definition and reference year

The SoEF statistics on FAWS area and growing stock are based on a common reference definition of FAWS^34^. However, the different interpretation of the reference definition or the use of different restrictions and related thresholds by each country caused the FAWS estimates in the international reporting to be, in practice, of limited comparability^34,50^. Moreover, such FAWS data are limited to statistics at national scale, while more detailed spatial information is needed to assess better and model the potential supply, and related costs, of woody biomass from the European forests.

Given these limitations, 22 European NFI institutions (AT, BG, CH, CZ, DE, DK, ES, FR, HU, IE, IS, IT, LT, LV, NL, NO, PL, PT, RO, SE, SK, SI) under the coordination of ENFIN identified and agreed upon a reference definition for the Forest Not Available for Wood Supply (FNAWS), which was accompanied by an explanation of the key terms, a harmonised list of restrictions to wood supply, and the comparison of the national and harmonised definitions^33^. Thus, 20 NFIs (excluding DK and FR) assessed, in a harmonised approach, the main restrictions to wood availability and quantified the forest area and biomass stock not available for wood supply^51,52^. The results are based on the same e-Forest estimator and NFI plot data used to calculate the harmonised biomass statistics described above, making the results on total standing forest biomass and the fraction not available for wood supply directly comparable.

Here, the harmonised FNAWS area and biomass of the 20 NFIs were subtracted from their total area and biomass stock to obtain the FAWS area and biomass statistics harmonised for reference definition and estimation method. However, these statistics were derived from NFI plot data acquired during different years that do not correspond across countries, ranging from 2002 to 2014. Thus, the FAWS statistics were further harmonised to a common reference year (i.e., 2020) using a linear adjustment factor, assuming that the change in FAWS area and biomass between the NFI year and the reference year 2020 was proportional to the change in the total forest area and biomass stock during the same period estimated with the CBM (as described above). For example, if the total national forest area (or biomass) increased by, e.g., 2% between the NFI year and the year 2020, the FAWS area (or biomass) was increased by the same amount.

### Forest available for wood supply: map

The forest area available for wood supply (FAWS area) was mapped by identifying and removing the areas not available for wood supply from the forest area map matching the harmonised statistics on total forest area (described above), while the biomass available for wood supply (FAWS biomass) was mapped by selecting the FAWS areas within the bias-adjusted biomass map (described above). In order to map the FAWS area, we used the harmonised information on the restrictions to wood availability provided by the NFIs of 20 countries, which quantified the forest area not available for wood supply for each restriction. These values were used as calibration data to produce restrictions to wood availability maps. Not all restrictions to wood availability can be mapped because there is no spatial information at European scale on forests used for recreational, cultural, or other non-harvesting purposes, or for protection against erosion or wind. Thus, we produced six maps that capture the main limitations to wood availability, namely the forest not available due to: steep slope, high altitude, protected areas, protected species, poor accessibility and low productivity.

The spatial distribution of these restrictions was obtained as follows. The forest areas located on too steep slopes or above the maximum altitude were identified using the European Digital Elevation Model (EU-DEM)^53^. Protected areas were defined as the areas classified as IUCN category I and/or II, and were mapped using the World Database on Protected Areas (WDPA)^54^. The protected species were mapped as the areas with the probability of species occurrence >10% according to the JRC European Atlas of Forest Tree Species^55^ and classified as the corresponding forest type (Broadleaf or Coniferous) according to the Copernicus Forest Type map^32^. The protected species indicated by the NFIs were cork oak (*Quercus suber L*.) and holm oak (*Quercus ilex L*.) in Portugal, and dwarf mountain pine (*Pinus mugo T*.), which however was already included in the altitude restriction because located in areas above the maximum altitude. Forests with poor accessibility were identified according to their distance to paved and unpaved roads, which were mapped using the OpenStreetMap database^56^. The forest areas with low productivity were identified using the kNDVI, a vegetation index highly correlated to the vegetation Gross Primary Productivity^57^. This index was computed from the red and near-infrared bands of the 16-day MODIS 250 m data (MYD13Q1 V6.1 product) using a value of 0.15 for the kNDVI sigma parameter. The kNDVI map representative of the year 2020 was obtained as the mean of the kNDVI for each 16-day period, resampled to 100 m, over the period 2018–2022 for Europe.

For each restriction, the threshold that defines the areas not available for wood supply was set separately for each country to consider the differences in forest management and legislation. The threshold was set to the value that, when applied to the restriction map, identifies an area corresponding to the forest not available for a given restriction according to the reference statistics. The thresholds were set at national scale (i.e., the same value is applied for the whole country) with the exception of SE and NO, where the threshold for unproductive forests was set separately for the Northern and the Southern parts of the countries, to account for the large ecological gradient.

The country-specific thresholds are relatively constant for altitude and productivity, as most countries indicated that the forests are not available for wood supply above 2000 m altitude or with a productivity below 1–2 m^3^/ha/year (corresponding to kNDVI values between 0.1–0.2). Similarly, the protected forests usually include the IUCN category I and/or II. Instead, the thresholds for other restrictions can vary considerably from country to country. For example, the maximum slope that allows harvesting is usually around 30 degrees but it varies from 20–25 degrees in flat countries to 40–45 degrees in mountainous countries. Similarly, the maximum distance to roads tends to be about 1000 m but it ranges from 500 m to 3000 m, according to the timber value and the technological capabilities of the harvesting systems used in the country. For the countries without information on the restrictions to wood availability, that is, the countries not included in the FAWS harmonisation study and where the SoEF database does not provide the FAWS area by restriction (AD, AL, BA, BE, CY, DK, EE, FI, GB, GR, HR, LI, LU, ME, MK, MT, RS), the thresholds were set using the corresponding values from the neighbouring country, or the average of neighbouring countries, that have similar ecological conditions and forestry systems (see Supplementary Information). The thresholds used for each parameter and country are provided in the data repository (see section on Data records).

### Forest increment: statistics

The growth rate of the forest, or forest increment, represents the “interest” that can be utilised without reducing the “capital” of growing stock, and thus it is an essential parameter to inform the sustainable use of forest resources. However, even more than for biomass stock, the increment data provided individually by the European NFIs and regionally compiled for international reporting (e.g., SoEF or FAO reports) are based on different approaches and definitions, adapted to national circumstances^9,58^. In this study, the increment data at sub-national scale of 10 countries representing 71% of the forest area of Europe were harmonised using a common definition and updated to the reference year 2015. For the countries not involved in this study, the increment statistics at national scale were obtained from the SoEF database for 25 countries representing 23% of the European forest area and, for the 3 countries (6% of European forest area) that did not report increment values in the SoEF, the statistics were derived from the CBM. Thus, we processed and compiled the available data on forest increment provided by the NFIs, the SoEF and the CBM to obtain a dataset on annual increment that is complete and harmonised, as much as possible, in terms of increment definition and reference year. For all European countries, the statistics provide the annual gross and net increment and the natural losses (i.e., the GAI, NAI and ANL) for both the forest and FAWS areas for the year 2015, estimating the missing values using linear adjustments.

#### Harmonisation of increment definition

The NFI institutions of AT, CH, DE, ES, FI, FR, IT, RO and SE, under the coordination of ENFIN, identified and agreed upon a reference definition of the forest increment and then, with the participation of CZ and PL but without CH, applied it to the national data using a common estimator and various harmonisation measures to account for the deviations between the national and harmonised definitions^59,60^. The harmonised gross increment (GAI) is defined as the average annual increment of living trees over the specified forest area during the period between two NFIs. It includes the growth components of survivor, ingrown, cut and mortality trees with a diameter at breast height (dbh) ≥7.5 cm, and refers to the over-bark increment of the stem from stump height to the top diameter of 7 cm. For broadleaves, the increment also includes large branches with a minimum diameter of 7 cm. The net increment (NAI) is computed as the gross increment (GAI) minus the natural losses (ANL), which in turn refers to the average annual losses related to trees that die during the period between two NFIs and remain unharvested in the forest.

The implementation of the harmonised definition and method considered the difference in sampling designs, applied a common dbh-threshold, and included specified tree parts and components of change. The harmonised GAI, NAI, and ANL are expressed in terms of volume (m^3^/year) over-bark at sub-national scale and refer to the same forest area and growth components. The impact of the harmonisation of the definition, computed as the difference between the harmonised and the national estimates divided by the national estimates, varied substantially among the countries, ranging from −11% to +12%.

#### Harmonisation of reference year

The NFI increment estimates of the 10 countries harmonised for definition and estimation method were usually obtained using the latest two completed NFI cycles and thus refer to different periods among countries, spanning between 1986 and 2020. Considering that the increment rates may change substantially in such a time frame because of changes in the forest area, age structure, mortality and harvest, these increment statistics were linearly adjusted to a common reference year using the time series of volume increment provided by the SoEF database. The reference year was set to the year 2015, because this is the latest reporting year currently available in the SoEF for increment. The forest and FAWS area provided by the NFIs were also adjusted to match the values reported by SoEF for the year 2015.

For six countries (CZ, FI, ES, PL, RO, SE), the temporal adjustment of the increment values was not necessary because their estimates were obtained from data acquired approximately between 2010 and 2020, and their estimates were considered representative of the mid-year 2015. For four countries (AT, DE, FR, IT), the adjustment was performed using a correction factor obtained as the ratio between the SoEF GAI value in 2015 and the average SoEF GAI value for the NFI reporting period, which quantifies the relative change of the GAI (in m^3^/ha/year) during this period. The correction factor was computed using the GAI data relative to FAWS rather than all forest area because it is reported more frequently in the SoEF and is considered more accurate than the increment for all forests, due to the larger density of field samples placed in productive and accessible forests. The correction factor for GAI was computed at national level and then it was multiplied to the harmonised GAI (m^3^/ha/year) of each country at sub-national level to update it to the year 2015. For example, the harmonised increment values of AT were obtained using the NFI data acquired during the period 2000 – 2009. According to the SoEF, the GAI of AT decreased by 2% between the period 2000–2010 and 2015, and thus the NFI harmonised GAI values were reduced by the same percentage to update them to the year 2015.

The ANL was considered as a relatively stable percentage of the GAI and, for the four countries for which we performed the temporal adjustment, it was adjusted to 2015 by multiplying the corrected mean (i.e., per ha) GAI for the ratio between the mean ANL and the mean GAI before correction. Then, the mean NAI was obtained as the difference between the GAI and the ANL. Lastly, the adjusted mean GAI, ANL and NAI (m^3^/ha/year) of each country were multiplied for the respective forest or FAWS area for the year 2015 reported by the SoEF to obtain the corresponding total increment values (m^3^/year).

The impact of the temporal synchronisation to the year 2015 of the mean increment values (m^3^/ha/year) was in the range of ±2%. Instead, the impact of the temporal synchronisation for the total increment values (m^3^/year) was usually larger, ranging from –6% to +10%, because it also included the adjustment in forest area to match the 2015 SoEF values. For the countries without the NFI harmonised estimates, the temporal synchronisation was not necessary because the increment data were obtained from the SoEF or from the outputs of CBM, which provide the increment values at national scale for the year 2015.

#### Gap filling for missing data

As for the other forest variables, the increment estimates refer to a certain forest definition and category (e.g., total forest area or FAWS area). In many cases, the mean increment is higher for FAWS than for total forest areas because the FAWS usually includes the most productive forests. In order to provide complete and, as far as possible, consistent data on the forest increment, we computed the gross and net increment both for the total forest land and for the FAWS area, estimating the missing values as follows. These estimated values have a larger uncertainty compared to the values estimated using appropriate national data, as they are derived using adjustment factors or values from neighbouring countries (see Supplementary Information).

Regarding the 10 countries with harmonised increment statistics, we first assessed their forest definition to attribute their values to the total forest area or to the FAWS area. All NFIs included in their values the productive and temporary unstocked forests, and excluded permanently unstocked forests (e.g., forest roads, firebreaks, forest depots), but there was some variability regarding the unproductive forests. The increment values of six countries (CZ, ES, FI, IT, PL, SE) included all protective and unproductive forests and were attributed to the total forest area, while the other four countries (AT, DE, FR, RO) excluded unproductive, inaccessible and protective forests without yield, and the increment values were attributed to the FAWS area. Even though the reported NFI forest classes did not match exactly the forest and FAWS categories (e.g., the FAWS area usually excludes also the protective forests with yield), the comparison with the areas reported in the SoEF confirmed that the attribution was appropriate because the forest and FAWS areas were comparable.

Then, the harmonised increment values for the missing category (forest or FAWS) were obtained as follows. If the country did not report increment data in the SoEF database for both categories and they presented a similar (i.e., < 15%) area (AT, FR, IT, PL), the mean increment (m^3^/ha/year) was simply considered equal for forest and FAWS, and the total increment (m^3^/year) was obtained by multiplying the mean increment for the respective (forest or FAWS) area. Instead, if the SoEF reported the increment for both forest and FAWS, the ratio between the two values was used as adjustment factor, as follows. If the harmonised missing value was for forest (i.e., DE, RO), the ratio between the SoEF forest and FAWS mean increment was multiplied to the harmonised value for FAWS, and vice-versa if the missing value was for FAWS (i.e., CZ, FI, SE). The correction was computed at national scale and applied to the increment data at sub-national scale. In the case of ES, the harmonised NFI data were provided for both forest and FAWS areas, and no further correction was needed.

For the countries without harmonised NFI statistics, the increment values at national scale were obtained directly from the SoEF database (AL, BE, BG, CH, CY, DK, EE, HR, HU, IE, IS, LI, LT, LU, LV, ME, MK, NL, NO, RS, SI, SK) or, for the EU countries where the SoEF did not report increment data, from the CBM outputs for the year 2015^21^ (GB, GR, PT). When the increment estimates were available only for forest or FAWS, the mean increment was simply considered equal for forest and FAWS if the forest and FAWS presented a similar (i.e., < 15% difference) area (GR, LU, LV, ME, SI). If the forest and FAWS had an area difference larger than 15%, the missing values were estimated using the data reported by the neighbouring country with the most similar forest conditions (AL, BG, IS, LI, LU, MK, RS). For example, if a country reported only the mean GAI, the mean NAI was estimated by multiplying the GAI with the ratio NAI/GAI of a similar country. Lastly, if a country did not report any value in the SoEF and is not included in the CBM (i.e., AD, BA, MT), all increment mean values were considered equal to those of a neighbouring country (or average of the neighbouring countries, if more appropriate) with similar forest conditions. The choice of the neighbouring countries with similar forest conditions used to produce each data record is reported in the Supplementary Information.

## Data Records

The biomass dataset provides statistics and maps of the forest biomass stock, its share available for wood supply and the forest increment for Europe. All data are freely accessible through Figshare^61^ (10.6084/m9.figshare.c.6465640).

The statistics at national and sub-national level are stored both as one Excel file (“Biomass Statistics (Excel)”) and two Shapefile (“Biomass Statistics (shp)” and “Increment Statistics (shp)”), which provide the biomass, FAWS and increment for different administrative or NUTS units. They provide: the forest area (ha), the forest area available for wood supply (FAWS) (ha), the forest area not available for wood supply (FNAWS) (ha), the forest aboveground biomass as AGB stock (t) and AGB/ha (t/ha), the forest aboveground biomass stock available for wood supply (BAWS) (t) and the forest aboveground biomass stock not available for wood supply (BNAWS) (t) for the year 2020; and the forest area (ha), the Forest area Available for Wood Supply (FAWS) (ha), and the Gross Annual Increment (GAI), the Annual Natural Losses (ANL), the Net Annual Increment (NAI) for the forest area and FAWS area for the period 2010–2020 (reference year: 2015) in units of volume per year (m^3^/year) and volume per hectare per year (m^3^/ha/year). The description of the content of the excel file is provided in the “ReadMe” spreadsheet within the file.

The maps are available in GeoTIFF format at the spatial resolution of 100 × 100 m in ETRS89 LAEA (EPSG:3035) coordinate reference system for the extent of Europe (see Fig. 1) for the forest area (“Forest Map 2020”), the forest area available for wood supply (“FAWS Map 2020”), the forest aboveground biomass density (t/ha) (“Biomass Map 2020”) and the forest aboveground biomass density available for wood supply (t/ha) (“BAWS Map 2020”).

The input data, calculations and assumptions used to produce each data record are provided in an additional Figshare repository^62^ (10.6084/m9.figshare.c.6787140.v6). The input data, calculations and assumptions used to produce the statistics on biomass, FAWS and increment are reported in the additional Excel files “Biomass Calculations (Excel)” and “Forest Increment Calculations (Excel)”. The country-specific thresholds used to produce the FAWS map are provided in Excel files “FAWS Map thresholds (Excel)”. The description of the content of the excel files is provided in the “ReadMe” spreadsheet within the files. The input data and processing steps used to produce the four maps are described in the R codes (see section below on Code Availability).

## Technical Validation

### Statistics

The harmonised statistics produced in this study were obtained using statistical inference from the NFI plot-level estimates based on harmonised definitions, which were then scaled to a common reference year using a modelling approach. The accuracy of the harmonised statistics may be affected by the validity of the estimator, the choice of the harmonised definitions and the type of modelling framework. The design-based estimator used in this study, called e-Forest, provides statistically-sound estimates of the forest variables and their precision is quantified by the design-based sampling error^37^. In general, the sampling errors of the harmonised estimates at national scale were small (see below) and comparable with the sampling errors based on the national definitions and estimators.

The statistics harmonised for definition were then updated to a common reference year using correction factors obtained from the CBM or linear models. The errors introduced in this step cannot be directly quantified but the reliability of the correction factors derived from the CBM is supported by the fact that this model was adapted to the specific European conditions, calibrated using country-specific data and used extensively for assessing the carbon dynamics of European forests (see^40–42^). Moreover, the temporal harmonisation had a limited effect on the estimates of biomass stock, FAWS and increment, because it changed them by an amount always smaller than 11% of their original value (see Methods). The use of different correction factors for forest, FAWS and FNAWS was not possible because the CBM does not distinguish FAWS and FNAWS areas.

Instead, the statistics for the countries not included in the harmonisation study were derived from the SoEF database or the CBM, and in such cases no information on their uncertainty is provided by the data source. However, the SoEF data were used only for a limited part of the European forest area, namely 8% for the biomass stock, 23% for the increment, 29% for the FAWS area, and 38% for the FAWS biomass. The CBM values were used only for the increment statistics of three countries, corresponding to 6% of European forest area. In fewer cases, some values were not reported in the SoEF database or the CBM and were estimated using approximations from the existing data. The missing values for FAWS and biomass corresponded to ≤1% of the area of European forests, but were more frequent for the increment (see below).

In order to assess the reliability of the reference statistics and support multiple uses of this dataset, an uncertainty value was assigned to each estimate according to the data source and method used to estimate the forest variables. The uncertainty label allows to filter the data according to the user needs and is equal to 0 (highest reliability) for harmonised NFI data, 1 for values derived from the SoEF or CBM, 2 for values estimated using data from the same country, and 3 for values estimated using data from other countries (lowest reliability). A detailed assessment of the uncertainty of each dataset is provided in the following sections.

#### Forest area and biomass

The sampling error of the harmonised biomass stock at national level, reported by the 26 countries involved in the harmonisation study, was always within 4% of their stock density except IS (20%), where the high error is due to the low number of plots and the low biomass of the country^28,29^. Similarly, the sampling error of the national forest area, reported by 16 countries, was within 4% of the estimated area, besides IS (11%).

The sampling error of the FAWS area and biomass was not reported explicitly, but it is expected to be somewhere within the sampling errors of the total forest area and biomass (i.e., ≤4%) and the errors for FNAWS. The sampling error for FNAWS, reported by seven countries involved in the harmonisation study (BG, HU, LT, LV, NL, PL, SI), was between 3% and 12% of their FNAWS area and between 2% and 9% of their FNAWS biomass density^51,52^. Considering that the sampling error is related to the sample size from which it is calculated, that the FNAWS area represents 13% of the European forest land, and that the number of forest plots is usually higher in FAWS than in FNAWS areas, the sampling error for FAWS area and biomass is expected to be lower than for FNAWS and more similar to the errors reported for the total forest area and biomass because of the larger amount of sample plots available in the FAWS.

#### Forest increment

The uncertainty of the forest increment varies substantially according to the data source (i.e., NFI, SoEF, CBM) and country but, in general, is likely higher than that of biomass stock because of the complexities associated with the estimation of this parameter and the assumptions used to perform the temporal harmonisation and gap-filling the missing values.

The NFI data of 10 countries harmonised for increment definition reported a sampling error for the GAI and NAI within 2% of the increment value at national level, while for the ANL it was in the range 3%–10%^59,60^. An additional uncertainty was introduced by the temporal harmonisation performed using the relative change of the SoEF data but, as mentioned above, this step was applied only to four countries and its impact on the estimates was small.

The uncertainty introduced by gap-filling the missing data depends on the country. In general, when the increment was reported only for forest but not for FAWS or vice-versa, the mean increment was simply considered equal for forest and FAWS. This assumption likely caused some underestimation when the growth rates of forest were applied to FAWS and overestimation in the opposite case, because the unproductive forests, excluded from FAWS, have a lower increment compared to productive forest sites. Considering the 18 countries reporting the NAI both for forest and FAWS in the SoEF database, it results that their mean NAI (m^3^/ha/year) within FAWS is on average 11% higher than that for total forest area. In our dataset, since the NAI values for FAWS were missing for 30% of the European forest area while the NAI value for forest were missing for 16% of the forest area, the gap-filling of missing data may have caused a net underestimation of the NAI increment of European forests.

The SoEF database does not provide error metrics, and the uncertainty of their increment values was tentatively assessed by comparing them with the corresponding NFI harmonised values for the 8 countries included in both datasets. In general, the SoEF values were higher than the NFI values, with an overall difference in the mean NAI of 12%. However, the difference varied substantially by country and in two cases it was larger than 20%, suggesting relevant differences in the data and methods employed to obtain the estimates. The analysis of the SoEF Country Reports also indicated that, in several cases, the reported NAI values are not derived from field measurements but by adjusting the national GAI data, using different approaches and variable levels of accuracy, possibly because of the difficulty to obtain accurate data on the ANL.

### Maps

Our dataset includes 4 maps, representing the forest area, the forest area available for wood supply, the forest biomass density (AGB/ha) and the forest biomass density available for wood supply. The reliability of the maps is assessed by comparing them with the corresponding reference statistics used for their calibration or adjustment, and is described in the following sections. We used the relative Mean Absolute Error (rMAE) (i.e., the Mean Absolute Error^63^ divided by the mean of the reference data) as a measure of divergence between the maps and the reference statistics.

#### Forest area

The harmonised forest area map was produced with a bias-adjustment approach that assures that the map matches the forest area provided by the reference statistics at the administrative scale. The original Copernicus Forest Type 2018 map (converted to a forest - non-forest map) and our bias-adjusted forest area map were compared with the reference statistics for Europe, including 38 countries divided in 289 administrative units. The original Copernicus map presented a rMAE of 10.8%, while this was only 0.3% for the bias-adjusted forest map, confirming that the adjusted map matched almost perfectly the reference statistics at their administrative scale. The bias adjustment corrects the systematic differences with the reference data but does not remove the random errors affecting the map accuracy at pixel level, for which we refer to the validation reports provided with the original Copernicus product^64,65^.

#### Forest biomass

As for the forest area map, the harmonised forest biomass density map was obtained with a bias-adjustment approach and was masked using the forest area map. The original and the bias-adjusted ESA CCI biomass maps were compared with the reference data for the 289 administrative units with harmonised statistics. The comparison was performed for the biomass stock (t) to account for the variable area of the administrative units. The original ESA CCI map presented a rMAE of 18.2%, while this was 0.3% for the bias-adjusted biomass map. Thus, the adjusted map matches the biomass density (t/ha), the biomass stock (t) and the major spatial patterns of the reference statistics at their administrative scale. Regarding the random errors affecting the map accuracy at pixel level, we refer to the uncertainty layer provided with the original ESA CCI biomass map^31^. We also note that the random errors may be reduced in the near future using, as input, a novel biomass map based on a sensor highly sensitive to the local variations in forest biomass (e.g., a map based on a lidar or P-band radar sensor), which would result in a bias-adjusted map matching the reference statistics at administrative scale and also providing a high pixel-level accuracy.

#### Forest area available for wood supply

The FAWS area map was calibrated using the reference statistics at national scale but, as indicated above, the restrictions to wood availability used in the reference data and in the map did not always correspond. Nonetheless, the two datasets provide comparable estimates at European scale: the FAWS area in Europe is 86% of the total forest area according to the reference statistics and 88% according to the map. The map matches well the statistics also at the country scale, with only 4 countries having a difference larger than 10%. When comparing the area identified by the reference statistics at national scale with the FAWS map, the rMAE is 2% for the FAWS area and 12% for the FNAWS area. The good match of the total area does not assure that the FAWS map also correctly identifies the spatial distribution of the FAWS areas at sub-national scale, which also depends on the mapping of the restrictions. For this reason, the FAWS area map was compared with the reference statistics at sub-national scale for 21 countries (159 administrative units) where the statistics and the map refer approximately to the same restrictions, and thus were comparable. The analysis showed that, compared to the statistics, the rMAE is 3% for the FAWS area and 17% for the FNAWS area.

#### Forest biomass available for wood supply

The FAWS biomass map was assessed by comparing the available biomass stock of the map with the reference statistics. The comparison was performed for the biomass stock (t) to account for the variable area of the administrative units. At European scale, the stock that is available for wood supply is 91% of the total standing stock according to the reference statistics and 90% according to the map. The map matches well the statistics also at the country scale, with 8 countries having a difference larger than 10%. As for the FAWS area map, the FAWS biomass map was compared with the reference statistics at sub-national scale for the 159 administrative units that presented comparable restrictions. Compared to the statistics, the rMAE is 5% for the FAWS biomass map and 40% for the FNAWS biomass map.

### Supplementary information

Supplementary Information

## Data Availability

The data presented in this study were produced as follows, and the respective codes are available in the additional Figshare repository^62^ 10.6084/m9.figshare.c.6787140.v6. All the statistics were derived from the NFI, SoEF or CBM input data and processed as described in the text. The input data and the processing steps performed to obtain the harmonised reference statistics are provided in the Excel files “Biomass Calculations (Excel)” and “Increment Calculations (Excel)”. The forest area map was obtained using the script “Forest_Map_code.R” (R version 4.0.2, 2020-06-22) to adjust the Copernicus 2018 Forest type map using the Copernicus 2018 Tree Cover map and match the reference statistics of forest area for 2020. The FAWS area map was obtained from the forest area map and six maps of restrictions to wood availability using the script “FAWS_Map_code.R” to map the FNAWS areas. The FNAWS area map was then used to mask the forest area map and obtain the FAWS area map using the raster calculator function of the QGIS software v. 3.22. The map of forest biomass density was obtained from the ESA CCI Biomass map for 2020 (v4) using the script “Biomass_Map_code.R” and the raster calculator function of the QGIS software to apply the bias-adjustment approach and match the reference biomass statistics for 2020 (as described in the text). The map of biomass available for wood supply was obtained by masking the bias-adjusted biomass map with the FAWS area map using the raster calculator function of the QGIS software.
