# Supplementary material for: Harmonised statistics and maps of forest biomass and increment in Europe
Source: Sci Data. 2024 Mar 6;11:274. doi: 10.1038/s41597-023-02868-8 (PMC10917757; doi:10.1038/s41597-023-02868-8)
Supplement: Supplementary file 1 — Supplementary Information [file 41597_2023_2868_MOESM1_ESM.pdf]

Table of Contents

Country-specific information 2

Country-specific information

This document provides detailed information regarding the data sources and the assumptions used in this study for each country that are not already provided in the main text of the article. The reference statistics referred to in this document are derived either from the SoEF 2020 database[22] or from NFI data harmonised for reference definition for biomass[28,29], FAWS[51,52] and increment[59,60]. The references for these sources are provided in the main text.

Albania (AL)

The data source is the SoEF 2020 database for all forest variables.

Forest map. The forest area reported in the SoEF for the year 2020 is 37% smaller than the area mapped by the Copernicus 2018 Forest Type map. The difference may be due to the classification of Other Wooded Land (OWL), which covers a large area and may be difficult to separate from forest. We adapted the Copernicus map to the SoEF value by removing the areas with lowest canopy cover.

FAWS map. The SoEF 2020 Country Report does not provide any information on the calculation of FAWS, and the productivity threshold was taken as the average of IT and RO, which NFIs provide specific data on FAWS restrictions.

Increment. The SoEF reports only the NAI in FAWS. The other increment values for FAWS and forest were estimated using proportions with the SoEF values reported for ME.

Andorra (AD)

The data source is the SoEF 2020 database. However, only the national forest area is reported in the SoEF, and the other forest variables are estimated using data from neighbouring countries.

Biomass stock. The forest biomass density of the country is considered equal to that of the neighbouring administrative unit ES513.

FAWS area and biomass. The percentage of FAWS in the country is considered equal to that of the neighbouring administrative unit ES513. Then, the FAWS area and biomass stock are obtained by multiplying the FAWS percentage with the forest area and the forest biomass stock, respectively.

FAWS map. The thresholds for the restrictions were derived from a neighbouring country (ES) and a country with similar physical geography (i.e., mountainous) (AT).

Increment. The increment per ha for forest and FAWS are considered equal to those of the neighbouring administrative unit ES513.

Austria (AT)

The data source is NFI harmonised data for all forest variables.

Forest area. The NFI harmonised forest area is almost identical to the SoEF forest area and to the area in the Copernicus 2018 Forest Type map, and refers to the NFI forest area excluding the shrublands (3.858 Mha).

FAWS area and biomass. The NFI values consider the protective forests with yield as FAWS and the protective forests without yield as FNAWS. The inaccessible forests belong to the category of protective forests without yield and thus are included in the FNAWS area. Since no ground

data are available in the inaccessible forests, their biomass density is estimated using the values of accessible protective forests without yield. The NFI also provided the area of the permanently unstocked areas in productive forests, which are included in the total forest area but are excluded from FAWS and FNAWS. Thus, in Austria, the forest area is equal to the sum of FAWS, FNAWS and permanently unstocked areas.

Biomass stock. The NFI harmonised biomass density values refer only to the productive and accessible forests (3.345 Mha) and, if applied to the total forest area, they would overestimate the national forest biomass stock because productive and accessible forests tend to have a higher biomass density than that of forest areas, which includes also unproductive forests. Since the area of productive and accessible forests is almost identical to the FAWS area (3.347 Mha), the harmonised biomass density values were multiplied to the FAWS area to estimate the FAWS biomass (or, BAWS). The biomass stock in FNAWS was also provided by the NFI harmonised data and the forest biomass stock was obtained by summing the FAWS biomass with the FNAWS biomass.

Increment. The NFI provided the harmonised increment for FAWS. The increment for forest areas (FAWS + FNAWS) was estimated considering that FNAWS are largely protective forests without yield (accessible and inaccessible), which have a much lower increment than FAWS. Currently, an estimate of the volume stock change in accessible protective forests without yield is available (0.9 m^3/ha/year for the period 2010-2015) and is approximately regarded as mean NAI per ha for FNAWS areas and used to compute the NAI (m^3/year) for FNAWS. The NAI of forest areas was thus computed by summing the NAI (m^3/year) of FAWS and FNAWS. Similarly, preliminary NFI estimates of the mean GAI and ANL per ha in FNAWS (1.5 and 0.6 m^3/ha/year) were used to compute the respective GAI and ANL (m^3/year) for FNAWS, and the GAI and ANL of forest areas were thus computed by summing the GAI and ANL (m^3/year) of FAWS and FNAWS. Increment data for the accessible protective forests without yield based on field re-measurements will be available in a few years.

Belgium (BE)
The data source is NFI harmonised data for forest biomass and the SoEF 2020 database for the FAWS area and biomass and for the increment.

Forest area. The forest area was derived from the data reported in the 2012 NFI (source: FISE, https://forest.eea.europa.eu/datacatalogue).

Biomass stock. The NFI harmonised forest biomass density values refer only to the productive forests but, given that the FAWS area represents 96% of the forest area, these values were applied to the total forest area to quantify the total biomass stock.

FAWS map. The SoEF Country Report indicates that the FNAWS areas are "strict forest reserves and forests physically not accessible", which can be identified using the restriction maps. The thresholds for the restrictions were calibrated on the basis of the values used in the neighbouring countries (NL, DE) and further adjusted to match the FAWS area reported by the SoEF.

Bosnia and Herzegovina (BA)
The data source is the SoEF 2020 database. However, only the national forest area and the biomass stock are reported in the SoEF, and the other forest variables are estimated using data from neighbouring countries.

Forest map. The forest area reported in the SoEF for the year 2020 is 35% smaller than the area mapped by the Copernicus 2018 Forest Type map. The difference may be due to the classification of Other Wooded Land, which covers a large area and may be difficult to separate from forest. We adapted the Copernicus map to the SoEF value by removing the areas with lowest canopy cover.

FAWS area and biomass. The percentage of FAWS in the country is estimated as the average of the neighbouring countries (HR, ME, RS). Then, the FAWS area and biomass stock are obtained by multiplying the FAWS percentage with the forest area and the forest biomass stock, respectively.

FAWS map. The thresholds for the restrictions were derived from countries located in the same region that present harmonised NFI data on FNAWS (BG, HU, IT, RO).

Biomass stock. The SoEF database reports a biomass stock for 2020 (86 t/ha) substantially lower than the value reported in the FAO FRA for 2015 (156 t/ha), which indicates that the FAO calculations for 2010 "confirm that biomass and carbon stock are higher than suggested in officially reported data" (https://fra-data.fao.org/assessments/fra/2020/). In absence of additional information and considering the biomass density of neighbouring countries (180 t/ha for HR, 117 t/ha for ME, 117 t/ha for RS), we provisionally estimate the national biomass density of BA as the average of the SoEF and FAO values (122 t/ha), and flagged this value with "high uncertainty" (uncertainty value: 3).

Increment. The increment per ha for forest and FAWS are considered equal to those of the neighbouring countries (HR, ME).

Bulgaria (BG)

The data source is harmonised data derived from Forest Management Plans for the forest and FAWS area and biomass, and the SoEF 2020 database for the increment.

Data source. Bulgaria does not have a NFI system and the data on biomass and FAWS are derived from the values provided by the forest management plans in correspondence of the INSPIRE 10 x 10 Km grid intersections (377 points). These reference values were used as a surrogate of field plots to derive national estimates using the harmonised (e-Forest) estimator. The Forest Management Plans refer to the period 2001 – 2014 and, in absence of additional information, in this study they were referred to the median year (2007). The FAWS and biomass estimates, produced at NUTS-3 level, are reported at NUTS-2 level to increase the number of reference values for each administrative unit. The limited number of reference values causes a larger uncertainty of the estimates, which is considered similar to the uncertainty of the SoEF values (uncertainty value: 1).

Biomass and FAWS. We note that the values reported in the SoEF in 2020 for forest biomass density (44 tons/ha) and FAWS area (52% of forest land) are much lower than the values estimated in this study for biomass (115 tons/ha) and FAWS (82%), which are in line with the values reported by neighbouring countries. The FAWS map matches the FAWS statistics.

Increment. The SoEF reports only the NAI in forest areas. The other increment values for FAWS and forest were estimated using proportions with the SoEF values reported for Romania.

Croatia (HR)

The data source is NFI harmonised data for forest biomass (recently recalculated), and the SoEF 2020 database for the FAWS area, the FAWS biomass and the increment.

Cyprus (CY)
The data source is the SoEF 2020 database for all forest variables.

Biomass stock. The harmonised NFI data refer to the biomass stocks in productive forests, which corresponded to only 48% of all forest land. Therefore, the SoEF data was used to estimate the biomass stock of all forest land (last reporting year for biomass is 2015).

FAWS. We note that the SoEF reports a FAWS area of only 24% of the forest land, while the FAWS map identifies a larger area (55% of forest land) using the restrictions thresholds indicated by neighbouring or similar countries.

Increment. The mean increment values in forest were not reported in the SoEF and, due to the lack of other data sources, they were considered equal to those for FAWS area.

Czech Republic (CZ)
The data source is NFI harmonised data for all forest variables.

FAWS area. The NFI reported that the FNAWS area in protected areas is probably overestimated and that in some cases (i.e., bark beetle calamity, change of species composition, reduction of non-native species) not negligible volume of wood is harvested and placed on the wood market. However, it was not possible to estimate the area of timber production in national parks. Conversely, the FNAWS area (according to the reference definition) excluded protected landscapes, where wood production is restricted but not significantly.

FAWS map. The stands of *Pinus mugo* are not available for wood supply but they are located in high altitude forests and therefore overlap with the restriction due to altitude (included in the FAWS map), and no specific mapping of this species was performed.

Denmark (DK)
The data source is NFI harmonised data for forest biomass and the SoEF 2020 database for the FAWS area and the increment.

FAWS area. Owing to the rather flat landscape in Denmark, restrictions regarding use of the land for wood supply is mainly due to setting aside land for biodiversity conservation or protection of landscapes. The Agency for Nature Protection is working on a consistent map of protected forest and other land in Denmark but such a map has not yet been finalised.

Estonia (EE)
The data source is the SoEF 2020 database for all forest variables.

Finland (FI)
The data source is NFI harmonised data for forest biomass and the SoEF 2020 database for the FAWS area and the increment.

Forest area. Since 1996, the NFI has used two parallel forest definitions: the national and the FAO definition, which differed by about 4 million ha in 2010. The national definition is based on tree growth and whether there are trees on the stands or not. The SoEF refers to the FAO definition and matches the forest area used by the NFI harmonised data to estimate the biomass stock in this study.

FAWS area. The Finnish NFI reports the forest area at subnational level by productivity classes but, since FAWS is due to various factors besides the productivity (e.g., protected areas), we used the SoEF data at national level as reference for forest area and FAWS.

FAWS map. The FAWS area reported in the SoEF (88% of the forest area) is larger than the value in our FAWS map (85%) because the SoEF value (see SoEF Country Report) considers not accessible and unproductive forests as FAWS while these areas are considered as FNAWS in our map. We note that the SoEF FAWS value is larger than that of neighbouring countries (82% in SE, 59% in NO), which consider unproductive forests as FNAWS.

France (FR)

The data source is NFI harmonised data for forest biomass and increment, and NFI data (according to the national definition) for the FAWS area.

FAWS area. The forest and FAWS area were derived from the annual French NFI Report (2008 - 2012) at subnational level (https://inventaire-forestier.ign.fr/spip.php?rubrique250). The FAWS area is not explicitly reported and it was considered equivalent to the "Area of Productive forests", which excludes the not accessible forests and present an area identical to the FAWS area reported in the SoEF.

Biomass stock. The NFI harmonised biomass density values for 2010 (2008 - 2012) refer only to the productive and accessible forests (15.419 Mha), which corresponds to the FAWS area (15.606 Mha), because the forests not available for wood supply were not inventoried on the ground. In general, the biomass density of productive and accessible forests tends to be slightly higher than the average biomass density of all forest areas, which also include unproductive forests. However, since in France the FAWS area corresponds to 95% of the forest land and includes the low-productive forests, the NFI harmonised biomass density values were applied both to the FAWS and the total forest area, as the resulting overestimation of biomass stock occurring on the FNAWS area (5% of forest land) was considered negligible.

Germany (DE)

The data source is NFI harmonised data for all forest variables.

Data source. The NFI harmonised data for biomass refer to the NFI of 2002 while the data for FAWS refer to the NFI of 2012. Since the forest area remains very similar between the two NFI cycles, the data are considered comparable.

Biomass stock. The NFI harmonised data for biomass exclude the not accessible forests but, as these forests cover a small area (0.05 Mha), their exclusion is considered negligible and the NFI biomass density values are applied to the total forest area.

FAWS area and map. The FAWS area is computed using the NFI data according to the national FNAWS definition because, in Germany, it includes more restrictions than the reference FNAWS definition. However, the FNAWS area according to the national definition (6% of forest area) is largely due to restrictions that cannot be mapped because they refer to social restrictions and management decisions, for which there is no spatial dataset. Thus, the FNAWS map identifies a smaller FNAWS area compared to the statistics (3% of forest area).

Greece (GR)

The data source is the SoEF 2020 database for biomass and FAWS and the CBM outputs for the increment.

Data source. Greece has performed only one NFI in 1992, and the forest variables reported by SoEF for the period 2000 – 2020 are not derived from field measurements but are estimates based on extrapolation of the 1992 data.

Forest map. The forest area reported in the SoEF for the year 2020 is 26% smaller than the area mapped by the Copernicus 2018 Forest Type map. The difference may be due to the classification of Other Wooded Land, which covers a large area and may be difficult to separate from forest. As for the other countries, we adapted the Copernicus map to the SoEF value by removing the areas with lowest canopy cover.

Biomass stock. The mean biomass density reported by the SoEF remains almost constant between 2000 (31.7 t/ha) and 2020 (31.8 t/ha). However, the biomass density of the neighbouring countries is more than double the value reported for Greece, suggesting that the SoEF value may underestimate the current biomass density. Existing biomass maps indicate that the biomass density of northern Greece is indeed higher than the SoEF value while the values for southern Greece are more similar to the SoEF value. For this reason, in this study a new reference value for northern Greece (GR5) was defined as the average of the biomass density of the neighbouring countries (AL, BG, MK, TR) (76.7 t/ha). Instead, the rest of the country (GR3, GR4, GR6) maintained the reference SoEF value (31.8 t/ha). This approach created a new reference biomass value for Greece.

Hungary (HU)

The data source is NFI harmonised data for biomass and FAWS and the SoEF 2020 database for the increment.

Biomass stock. The NFI harmonised biomass density values refer only to the productive and accessible forests (1.83 Mha) and, if applied to the total forest area (2.14 Mha), they would overestimate the national forest biomass stock because productive and accessible forests tend to have a higher biomass density than that of forest areas, which includes also unproductive forests. Thus, the harmonised biomass density values were multiplied to the FAWS area to estimate the FAWS biomass (or, BAWS). The biomass stock in FNAWS was also provided by the NFI harmonised data and the forest biomass stock was obtained by summing the FAWS biomass with the FNAWS biomass.

Iceland (IS)

The data source is NFI harmonised data for biomass and FAWS and the SoEF 2020 database for the increment. Both datasets provide data only at national level. The sampling errors related to the estimates of FAWS and biomass are, in percentage, higher than those of other countries because of the low biomass density and the limited number of plots used to estimate the restrictions to FAWS.

Forest area. The harmonised NFI forest area was computed through plot proportion, and included the plots with the centre point within forest but excluded the plots with the centre point outside forest. This selection criteria of the field plots is one of the reasons for the difference between the harmonised forest area reported by the NFI and the SoEF with a country specific approach using all fractions of plots defined inside forest.

FAWS map. The NFI statistics identify a larger FNAWS area compared to the FNAWS map because the main restrictions to wood availability (“too crooked stems”) could not be mapped for lack of related maps.

Increment. The SoEF reports the NAI in forest and FAWS. The GAI and ANL values for FAWS and forest were estimated using proportions with the SoEF values reported for NO.

Ireland (IE)
The data source is NFI harmonised data for biomass and FAWS and the SoEF 2020 database for the increment.

Forest area. The area correction factor used to update the forest area from the NFI year (2006) to the year 2020 (about 1% increase per year) is higher than the value of other countries because of the large afforestation activities performed in Ireland during the last decades.

Biomass stock. The input NFI data, referring to the year 2006 and to the harmonised biomass definition, were updated to 2020 using a biomass change factor derived from the NFI national time-series data on carbon stock, recently recalculated using consistent and recent equations. FAWS area and biomass. The NFI time-series data indicates that the change rate between the NFI year (2006) and 2022 in the FAWS area is different from the change rate in the FNAWS area, as there is a larger increase in FNAWS than in FAWS areas. The same applies to the FAWS and FNAWS biomass. Therefore, the 2006 NFI harmonised data on FAWS and FNAWS area and biomass were updated to 2020 using change rates derived from the NFI time-series data.

FAWS map. The NFI statistics identify a larger FNAWS area compared to the FNAWS map for the following reasons. First, the two main restrictions to wood availability ("waterlogged areas" and "too crooked stems") could not be mapped for lack of related maps. Second, the "Protected areas" could not be mapped because the IUCN map does not identify any Protected area in category I and II in Ireland. Third, the area related to the restriction "too little yield" estimated in the map using the kNDVI value for unproductive forests was smaller than the area reported by the NFI statistics.

Italy (IT)
The data source is NFI harmonised data for forest biomass and the increment and the SoEF 2020 database for the FAWS area and biomass.

FAWS area and biomass. The FNAWS area estimated by the NFI harmonised data (0.6 Mha) is substantially lower than the values derived from the SoEF (1.1 Mha). The difference is due to the fact that the NFI harmonised data are derived from the 3rd phase plots (about 7,000 plots) because they provide information on the type of restriction to FAWS. Instead, the SoEF FAWS value is based on the 2nd phase plots (about 30,000 plots), which do not report information on the restrictions. As indicated in the NFI report, the FNAWS is under-represented in the 3rd phase plot sub-sample because several FNAWS plots are inaccessible and therefore not surveyed in the third phase, causing an unavoidable underestimate of the FNAWS area and biomass. For this reason, in this study we used the SoEF data to estimate the FAWS area and biomass for 2020, and these values are available only at national level.

Latvia (LV)
The data source is NFI harmonised data for biomass and FAWS and the SoEF 2020 database for the increment. Both datasets provide data only at national level.

FAWS area. The NFI harmonised data includes only environmental considerations in the estimation of the FAWS area, considering that economical or social restrictions are included within the environmental restrictions. The FNAWS area estimated in the NFI is about twice the FNAWS area derived from SoEF data, and this difference is due to different estimation methods and the use of different NFI cycles.

FAWS map. The FAWS map includes economic restrictions but the mapped FNAWS area is smaller than the NFI value because the maps of protected areas (IUCN cat. II) identifies a smaller FNAWS area compared to the NFI reported values for the same restrictions.

Liechtenstein (LI)
The data source is the SoEF 2020 database for all forest variables.

Increment. The SoEF reports only the NAI in FAWS. The other increment values for FAWS and forest were estimated using proportions with the SoEF values reported for CH.

Lithuania (LT)
The data source is NFI harmonised data for biomass and FAWS and the SoEF 2020 database for the increment.

FAWS area. The FAWS area is computed using the NFI data according to the national FNAWS definition because, in Lithuania, it includes more restrictions than the reference FNAWS definition (i.e., Natura 2000, soil protection and low profitability).

FAWS map. The FAWS map identifies a smaller FNAWS area compared to NFI value because the area of protected areas reported by the NFI is larger than the area identified by the map of protected areas (IUCN cat. II).

Luxemburg (LU)
The data source is the SoEF 2020 database for all forest variables. The FAWS biomass is not reported in the SoEF and it is estimated as the average of the neighbouring countries (HR, ME, RS).

FAWS map. The FAWS map identifies a larger FNAWS area compared to value derived from the SoEF because the mapped area of the protected area (IUCN cat. I) is larger than the SoEF total value.

Increment. The SoEF reports only the NAI in FAWS. The GAI in FAWS is estimated using the relation GAI/NAI reported for BE (neighbouring country with national data), and the mean increment values in forest are equal to those for FAWS because the FAWS area is similar (> 85%) to the forest area.

Malta (MT)
The data source is the SoEF 2020 database. However, only the national forest area is reported in the SoEF, and the other forest variables are estimated using data from neighbouring countries. The FAWS area and biomass are estimated using percent FAWS area and the FAWS biomass density of GR; the biomass stock is estimated using the average biomass density of southern Greece (GR3, GR4, GR6); the increment per ha is considered equal to that of CY.

Montenegro (ME)
The data source is the SoEF 2020 database for all forest variables.

The Netherlands (NL)
The data source is NFI harmonised data for biomass and FAWS and the SoEF 2020 database for the increment.

FAWS area. The FAWS area is computed using the NFI data according to the national FNAWS definition because, in the Netherlands, it includes the national strict reserves that are not considered in the harmonised FNAWS definition, which area is comparable with that of the IUCN reserves.

FAWS map. Most of the NFI FNAWS area (55%) refers to restrictions that cannot be mapped (i.e., management decision and cultural use of the forest) and thus the FAWS map provides a smaller FNAWS area compared to the NFI value. Also, the NFI data consider the accessibility restriction according to the difficulty in the field data collection, which may differ from the factors considered in the FAWS map, namely the distance to roads and slope.

North Macedonia (MK)
The data source is the SoEF 2020 database for all forest variables.

Increment. The SoEF reports only the GAI in FAWS. The other increment values for FAWS and forest were estimated using proportions with the SoEF values reported for ME.

Norway (NO)
The data source is NFI harmonised data for biomass and FAWS and the SoEF 2020 database for the increment.

FAWS area. The restrictions to wood availability affects a large part of the forest area of Norway, with the FNAWS area occupying 41% of the total forest land. The main restrictions are low productivity or a high distance from the roads.

FAWS map. Almost all restrictions used to estimate the NFI harmonised FAWS area can be mapped. The thresholds are constant at national level for all restrictions besides that for the productivity restriction (too little yield for wood supply). A single productivity threshold for the whole country resulted in large overestimation of FNAWS area in the north and underestimation in the south. Instead, the use of two different thresholds for the north-west region (NUTS-2 units NO41 - NO73) and the south region (NO11 - NO34) allowed us to better identify unproductive forests. This may be due either because in the north the productivity is associated with other environmental constraints that further limit the wood production, or because the productivity index presents different sensitivity to identify unproductive forests between the south and the north of the country. Still, the FAWS map identified a smaller unproductive area, and thus a smaller FNAWS area, compared to the NFI value.

Poland (PL)
The data source is NFI harmonised data for all forest variables.

FAWS area and map. The NFI harmonised data for FAWS identify various types of protected areas for environmental reasons (national parks, natural reserves, priority habitats, genetic reserves) but the analysis of the harvesting intensity in most protected forests revealed that it is comparable with that in forests without protection. Instead, the FAWS map considers as FNAWS only the strict natural reserves (i.e., IUCN cat. I and II) and therefore identifies a FNAWS area smaller than the NFI reference value. The accessibility restrictions are included in the NFI FNAWS data according to the harmonised definition and excluded in the national definition, while the military camps, which cover a large forest area (150,000 ha) are included in the FNAWS national definition but excluded in the harmonised definition.

Portugal (PT)

The data source is NFI harmonised data for biomass and FAWS and the CBM outputs for the increment.

Forest area and biomass. The input NFI data used in this study of forest and FAWS area and biomass stocks were recently recalculated by the NFI and are in line with the SoEF values. These values refer to the mainland for the year 2015, thus excluding the islands of Madeira and Azores. The biomass values include all aboveground tree compartments except the stump, and were updated to the year 2020 using the approaches described in the text.

FAWS area and map. The NFI FAWS data used in this study considers three restrictions (slope, protected areas, protected species). The protected species are cork oak (*Quercus suber* L.) and holm oak (*Quercus ilex* L.), which cannot be harvested.

Increment. The mean increment values in FAWS were not reported in the SoEF and, due to the lack of other data sources, they were considered equal to those for forest area.

Romania (RO)

The data source is NFI harmonised data for all forest variables.

FAWS map. The NFI harmonised FAWS area includes several restrictions that cannot be mapped for lack of appropriate spatial data and, therefore, the FAWS map identifies a larger FNAWS area compared to the NFI statistics. However, the difference between the FAWS map and the statistics is moderate because the majority of the FNAWS area is due to two restrictions that are considered in the FAWS map, namely slope (66% of FNAWS area) and protective forest for soil (10% of FNAWS area), which is highly correlated to high slopes.

Serbia (RS)

The data source is NFI harmonised data for biomass stock and FAWS area and the SoEF 2020 database for the FAWS biomass and increment.

Forest area and biomass. The data on forest area and biomass stock of the NUTS units RS11, RS12, RS21, RS22 were derived from the NFI harmonised data for reference definitions while the data of RKS (Kosovo) follow the national definition and were derived from: Tomter et al., 2013. Kosovo National Forest Inventory 2012. Kosovo Ministry of Agriculture, Forestry and Rural Development/Norwegian Forestry Group.

FAWS area and biomass. The FAWS area is not reported in the SoEF and it was estimated at national level using the average value of FAWS area reported in the following two documents: Vauhkonen et al., 2019, harmonised projections of future forest resources in Europe (https://doi.org/10.1007/s13595-019-0863-6); Vidal et al. (Editors), 2016, National Forest Inventories - Assessment of Wood Availability and Use (https://doi.org/10.1007/978-3-319-44015-6). The FAWS biomass was estimated multiplying the biomass stock (from the NFI data) with the fraction of GSV available for wood supply for Serbia provided by the SoEF 2020 database.

Increment. The SoEF reports only the NAI in forest areas. The other increment values for FAWS and forest were estimated using proportions with the SoEF values reported for Romania.

Slovakia (SK)

The data source is NFI harmonised data for biomass and FAWS and the SoEF 2020 database for the increment.

FAWS area. The NFI reports that the number of field plots acquired in FNAWS areas (123) is rather low to satisfactorily estimate FNAWS area and biomass. The main restriction is "Protective forest" that refers to forests on extremely unfavourable sites where forest management is limited.

Slovenia (SI)
The data source is NFI harmonised data for biomass and FAWS and the SoEF 2020 database for the increment.

FAWS area. The main restriction to FAWS are protective and protection forests. The NFI indicates that the protection function is identified on expert opinion and may be due to various factors, such as forests on high altitude to maintain the tree line, on steep slopes to protect from soil erosion, along river banks for water regulation and land protection, for protection from strong winds, or to protect highly biodiverse areas. The threshold for the slope restriction varies according to the local harvesting techniques and logging technology, which varies between the alpine regions in the northwest and hill regions in the southeast. Thus, each forest is evaluated individually and the FNAWS is the combination of terrain characteristics.

FAWS map. Since the protection forests are mostly due to the combination of slope, erosion and accessibility, the FNAWS areas are mapped using the restrictions of slope, altitude and distance to roads. The FNAWS map also includes the productivity restriction, which is not considered in the FAWS statistics.

Spain (ES)
The data source is NFI harmonised data for all forest variables (Ministerio para la Transición Ecológica y el Reto Demográfico). The statistics include the Canary Islands (ES701, ES702) while the maps exclude them.

Forest area. The NFI estimates the forest area using the Spanish Forest Map (scale 1:50,000 for 1998-2007 and 1:25.000 since 2008) produced by photo-interpretation using remote sensing as auxiliary data and including field visits. Currently, the national land use classification system and the NFI harmonised data and SoEF 2020 refer to the forest land according to the FAO definition including agro-silvo pastoral systems with tree cover up to 10%.

Forest map. The Copernicus 2018 Forest Type map identifies a forest area 15% smaller than the value reported by the NFI harmonised data and the SoEF. The difference may be due to the classification of agro-silvo pastoral areas. The forest map matches the forest area reported by the NFI harmonised data.

Biomass stock. The input NFI harmonised data for biomass stock in forest, FAWS and FNAWS areas were recently recalculated using improved allometric equations by the Spanish NFI. These estimates, referring to the NFI3 (1997-2007), were provided with a specific reference year for each NUTS3 administrative unit (Province), and were updated to the year 2020 using a correction factor specific for each NUTS unit.

FAWS area and map. Erosion and slope are the most important factors limiting the wood supply from Spanish forests. The NFI harmonised data reported that, in Spain, erosion and slope occurred mostly on different areas, with small overlap. Therefore, the FAWS map includes the slope restriction but does not properly represent the erosion restriction. Instead, the FAWS map includes the productivity restriction while this is not considered as a relevant FAWS restriction.

Sweden (SE)
The data source is NFI harmonised data for all forest variables, recently updated to also include the forest areas in the alpine ecological zone.

Forest area. The NFI harmonised data employed the FAO forest definition, with the exception that also productive forests with areas < 0.5 ha can be included in the forest area.
FAWS area. The NFI harmonised data identify that FNAWS covers 20% (5.7 Mha) of the forest land, while they represent 30% (8.4 Mha) according to the SoEF. This difference is partly due to the fact that the SoEF includes in the FNAWS the forests not available because of owner decisions (approx. 1 Mha), which instead are excluded in the NFI harmonised data because this information is not provided by the field plots.

FAWS map. The FAWS map considers the restrictions related to altitude, slope and distance to roads, which are excluded from the NFI harmonised FAWS data. Similarly to Norway, the productivity restriction was mapped at sub-national level because the use of a single productivity threshold for the whole country resulted in the overestimation of the FNAWS area in the north and underestimation in the south. Instead, using a threshold for the north (NUTS-2 unit SE33) and the south (SE11 – SE32) allowed us to better identify unproductive forests.

Switzerland (CH)
The data source is NFI harmonised data for biomass and FAWS and the SoEF 2020 database for the increment.

FAWS map. The FAWS map includes the altitude, slope and productivity restrictions, which are excluded in the NFI harmonised data, and thus identifies a larger FNAWS area than the NFI statistics. Since the logging operation uses hauling cables in mountain areas, the slope restriction was set to exclude only forests on extremely steep terrain (>45 degree). The distance to roads is considered both in the FAWS statistics and map, but the FAWS statistics compute the distance considering also the height difference into account while the FAWS map considers only the horizontal distance.

United Kingdom (GB)
The data source is the SoEF 2020 database for biomass and FAWS and the CBM outputs for the increment.

FAWS area. The SoEF reports that all forests are FAWS, but also acknowledges that some areas are FNAWS (e.g., protected forest areas) even though these areas cannot be quantified. Thus, the FAWS map considered six restrictions (as for all other countries) and set the restriction thresholds identified in the neighbouring countries (BE, DK, FR, NL).
